# Unbalanced Regulation of Sec22b and Ykt6 Blocks Autophagosome Axonal Retrograde Flux in Neuronal Ischemia–Reperfusion Injury

**DOI:** 10.1523/JNEUROSCI.2030-21.2022

**Published:** 2022-07-13

**Authors:** Haiying Li, Xiang Li, Zhongmou Xu, Jinxin Lu, Chang Cao, Wanchun You, Zhengquan Yu, Haitao Shen, Gang Chen

**Affiliations:** Department of Neurosurgery & Brain and Nerve Research Laboratory, The First Affiliated Hospital of Soochow University, Suzhou, Jiangsu Province 215006, China

**Keywords:** autophagic flux, cerebral ischemia-reperfusion injury, neuron, Sec22b, Ykt6

## Abstract

Cerebral ischemia–reperfusion (I/R) injury in ischemic penumbra is accountable for poor outcome of ischemic stroke patients receiving recanalization therapy. Compelling evidence previously demonstrated a dual role of autophagy in stroke. This study aimed to understand the traits of autophagy in the ischemic penumbra and the potential mechanism that switches the dual role of autophagy. We found that autophagy induction by rapamycin and lithium carbonate performed before ischemia reduced neurologic deficits and infarction, while autophagy induction after reperfusion had the opposite effect in the male murine middle cerebral artery occlusion/reperfusion (MCAO/R) model, both of which were eliminated in mice lacking autophagy (Atg7^flox/flox^; Nestin-Cre). Autophagic flux determination showed that reperfusion led to a blockage of axonal autophagosome retrograde transport in neurons, which then led to autophagic flux damage. Then, we found that I/R induced changes in the protein levels of Sec22b and Ykt6 in neurons, two autophagosome transport-related factors, in which Sec22b significantly increased and Ykt6 significantly decreased. In the absence of exogenous autophagy induction, Sec22b knock-down and Ykt6 overexpression significantly alleviated autophagic flux damage, infarction, and neurologic deficits in neurons or murine exposed to cerebral I/R in an autophagy-dependent manner. Furthermore, Sec22b knock-down and Ykt6 overexpression switched the outcome of rapamycin posttreatment from deterioration to neuroprotection. Thus, Sec22b and Ykt6 play key roles in neuronal autophagic flux, and modest regulation of Sec22b and Ykt6 may help to reverse the failure of targeting autophagy induction to improve the prognosis of ischemic stroke.

**SIGNIFICANCE STATEMENT** The highly polarized architecture of neurons with neurites presents challenges for material transport, such as autophagosomes, which form at the neurite tip and need to be transported to the cell soma for degradation. Here, we demonstrate that Sec22b and Ykt6 act as autophagosome porters and play an important role in maintaining the integrity of neuronal autophagic flux. Ischemia–reperfusion (I/R)-induced excess Sec22b and loss of Ykt6 in neurons lead to axonal autophagosome retrograde trafficking failure, autophagic flux damage, and finally neuronal injury. Facilitated axonal autophagosome retrograde transport by Sec22b knock-down and Ykt6 overexpression may reduce I/R-induced neuron injury and extend the therapeutic window of pharmacological autophagy induction for neuroprotection.

## Introduction

Autophagy is a core molecular pathway for the preservation of cellular and organismal homeostasis (Klionsky et al., 2021). Commensurate with the multipronged layers of autophagy initiation, changes in autophagic flux affect the pathogenesis of brain disorders, such as Alzheimer's disease and Parkinson's disease (Menikdiwela et al., 2020). Autophagy defects are particularly detrimental for postmitotic cells (e.g., neurons, cardiomyocytes, memory T cells), largely linked to their accrued demands for long-term proteostasis (Pearson and Walker, 1968; Scrivo et al., 2018). A few general concepts emerging from the abundant preclinical literature point to autophagy modulating interventions as promising approaches to prevent or mitigate phenotypic anomalies of human diseases (Klionsky et al., 2021).

Stroke is a debilitating disorder with significant annual mortality and morbidity rates worldwide (Selvaraj et al., 2021). Autophagy is extensively observed in experimental ischemic stroke (Zhang et al., 2013; Jiang et al., 2018). Most of the reports attest to a protective role of autophagy in stroke by removing damaged proteins and organelles (Carloni et al., 2008). We previously reported that brain microvascular endothelial cell (BMVEC) autophagy induced by rapamycin and lithium carbonate pretreatment exerted a protective effect on integrity during experimental cerebral ischemia–reperfusion (I/R) injury (H. Li et al., 2013). However, these observations are difficult to reconcile with equally compelling evidence demonstrating autophagy activation as a double-edged sword in ischemic stroke (Koike et al., 2008; Wen et al., 2008; Wei et al., 2012; Ginet et al., 2014; Luo et al., 2017; Feng et al., 2018). Autophagy inhibition by 3-methyladenine (3-MA) at 24 h before reperfusion induced an increase in neuronal death, while 3-MA treatment at 48–72 h after reperfusion significantly inhibited neuronal death (Shi et al., 2012). It has also been reported that when rapamycin and 3-MA treatments were performed 20 min before hypoxia-ischemia, rapamycin promoted autophagy and attenuated brain injury, while 3-MA inhibited autophagy and enhanced neuronal death (Puyal et al., 2009). However, when given at 4 h after ischemia (2.5 h after reperfusion), 3-MA administration strongly reduced lesion volume (Puyal et al., 2009).

Autophagy induction can only be beneficial when autophagic flux is intact (H. Li et al., 2013). Intact autophagic flux is essential for cell homeostasis, especially in terminally differentiated and nondividing neurons (Komatsu et al., 2006). It has been reported that impaired autophagic flux is associated with neuronal cell death after traumatic brain injury (Sarkar et al., 2014). There is a dramatic upregulation of microtubule-associated protein 1 light chain 3 (LC3) and beclin-1 in the brain in a rat transient cerebral ischemia model, indicating autophagy activation following I/R (Rami et al., 2008). However, the accumulation of autophagy-associated proteins after ischemia may correspond to the failure of autophagic flux rather than autophagy induction (Liu et al., 2010). In addition, it is essential to understand the traits of autophagy in the poststroke brain for possible applications in autophagy-based therapies for stroke.

Because of the highly polarized architecture with axons in neurons, neuronal autophagy possesses unique localizations of lysosomes and autophagosomes, in that lysosomes are mainly located in the cell soma, but autophagosomes are preferentially generated at the neurite tip (Maday et al., 2012; T. Wang et al., 2015). Following biogenesis, distal autophagosomes undergo retrograde transport toward the cell soma. The damaged axonal mitochondria were retrogradely transported to neuronal soma and underwent autophagic clearance (Zheng et al., 2019). Autophagosome retrograde trafficking may serve as a surveillance mechanism for routine maintenance of the axon (Maday and Holzbaur, 2012) and is a constitutive process for autophagic flux in neurons. Atg7 acts as an E1-like enzyme for both the LC3-conjugation and Atg12-conjugation systems, which are essential for autophagy (Ravikumar et al., 2010; Collier et al., 2021). Central nervous system-specific Atg7 conditional knock-out mice (Atg7^flox/flox^; Nestin-Cre; Komatsu et al., 2006) have been made available for assessing the physiological and pathologic functions of autophagy in the brain (Komatsu et al., 2006; C.W. Chen et al., 2012).

In this study, we found that cerebral ischemia induces neuronal autophagy, while reperfusion damages the integrity of autophagic flux, and Sec22b and Ykt6 are involved by regulating axonal autophagosome transport.

## Materials and Methods

### Animals

All animal experimentation was conducted in accordance with SfN's Policies on the Use of Animals and Humans in Neuroscience Research. All the animals were randomly assigned to experimental groups using a computer random number generator.

Sprague–Dawley (SD) rats (weight: 250–300 g, age 8–10 weeks) were purchased from Zhaoyan New Drug Research Center Co, LTD. Adult C57BL/6 mice (weight: 25–30 g, age 8–10 weeks) were purchased from the Animal Center of the Chinese Academy of Sciences. Atg7^flox/+^ mice [B6. Cg-Atg7<tm1Tchi> (RBRC02759)] mice were purchased from Nanjing Biomedical Research Institute of Nanjing University (National Resource Center For Mutant Mice) and originated from the RIKEN BRC through the National Bio-Resource Project of the MEXT, Japan (Komatsu et al., 2005), and Nestin-Cre transgenic mice [B6. Cg-Tg(Nes-cre)1Kln/JNju] were purchased from Nanjing Biomedical Research Institute of Nanjing University. Atg7^flox/+^ mice were inbred to produce Atg7^flox/flox^ mice, which were then bred with Nestin-Cre transgenic mice to produce Atg7^flox/+^; Nestin-Cre mice and Atg7^flox/flox^; Nestin-Cre mice. Mice were housed in a pathogen-free facility and were used at 10 weeks after birth (weighing ∼20 g). Male animals were used in the experiment, except for the part that indicated the use of female animals.

### Murine middle cerebral artery occlusion/reperfusion (MCAO/R) model

A murine MCAO/R model was established with nylon filaments (A4-263650 for rats and A4-162050 for mice, Beijing Cinontech Co, Ltd.) as described previously (Z. Wang et al., 2017). Rats received recanalization after 2 h of MCAO, and mice received recanalization after 1 h of MCAO. Cerebral blood flow was monitored by a laser-speckle imaging system (RFLSIIII, RWD Life Technology Co) for mice and a Doppler flowmeter (moorVMS-LDF, Moor Instruments) for rats.

### Cell cultures

hCMEC/D3 cells were obtained from the Cell Bank of the Chinese Academy of Sciences. hCMEC/D3 cells were cultured as routine in 1640 medium (HyClone, SH30809.01B) supplemented with 10% heat-inactivated fetal bovine serum and maintained at 37°C under humidified conditions and 5% CO_2_.

Primary cortical neurons were extracted from 16-d-old mouse embryos. First, the whole brains of fetal mice were extracted, and the cerebellum and brainstem were removed. In precooled PBS, the blood vessels and arachnoids were microscopically dissected from the surface of the bilateral cortex. Next, the cortical tissue was digested using 0.25% trypsin at 37°C for 5 min, washed with PBS three times, and blown into the cell suspension. The suspension was centrifuged at 500 × *g* for 5 min. The dissociated neurons were plated at a density of 1 × 106 cells/well on a 6-well plate and at 2 × 10^5^ cells/well on a 12-well plate (Corning) coated with poly-D-lysine (Thermo Fisher Scientific). The neuron culture medium included neurobasal medium, B27 supplement, 0.5 mm glutamine, and penicillin/streptomycin (Thermo Fisher Scientific). The culture environment was maintained at 37°C with humidified 95% air and 5% CO_2_.

### Establishment of *in vitro* oxygen-glucose-deprivation/reoxygenation (OGD/R) model

To mimic I/R *in vitro*, hCMEC/D3 cells and neurons were exposed to OGD/R. hCMEC/D3 cells were maintained in low-serum (2% FBS) and glucose-free medium in a humidified incubator containing 95% N_2_ and 5% CO_2_ at 37°C for 1 h. For reoxygenation with glucose reintroduction, cells were again cultured in standard medium and placed in an incubator containing 70% N_2_, 25% O_2_, and 5% CO_2_. For the *in vitro* OGD/R model of neurons, neurobasal medium was replaced with DMEM (Invitrogen), and cells were transferred to a 5% CO_2_ and 95% N_2_ atmospheric incubator for 1 h at 37°C. After that, neurons were cultured in neurobasal medium again and maintained in a 5% CO_2_ atmospheric incubator.

### RT-PCR

Genotyping was performed after extracting genomic DNA from tail snips using a DNeasy Blood & Tissue kit (QIAGEN Inc., catalog #69504). PCR analyses of the DNA were performed to detect Cre and floxed-Atg7 alleles using corresponding primer sets with standard conditions (Cre: 5 min at 95°C, 30 s at 94°C, 35 s at 58°C, 45 s at 72°C for 35 cycles, and 3 min at 72°C; Atg7flox/flox: 5 min at 95°C, 30 s at 94°C, 30 s at 60°C, 45 s at 72°C for 35 cycles, and 5 min at 72°C). The sequences of PCR primers for genotyping Atg7^flox/flox^ mice (Kang et al., 2017) were as follows: two forward primers, 5′-TGGCTGCTACTTCTGCAATGATGC-3′ and 5′-ATTGTGGCTCCTGCCCCAGT-3′, and a reverse primer, 5′-CAGGACAGAGACCATCAGCTCCAC-3′. The 460-bp PCR product was detected in wild-type (WT) mice, and the 550-bp PCR product was detected in homozygous Atg7^flox/flox^ mice. In heterozygous mice (Atg7^flox/-^), both 460- and 550-bp PCR products were detected. The Cre transgene was detected by PCR using the primers 5′-ATTTGCCTGCATTACCGGTC-3′ (forward) and 5′-ATCAACGTTTTCTTTTCGG-3′ (reverse) to amplify a 350-bp DNA product.

### Drug treatment

Rapamycin was dissolved in DMSO at a stock concentration of 0.01 m, while lithium carbonate was dissolved in double-distilled water at stock concentrations of 0.01 m. In *in vivo* experiments, rapamycin (150 µg/kg body weight) and lithium carbonate (20 mg/kg body weight) were injected via the tail vein at 0.5 h before MCAO or 4 h after MCAO/R. In *in vitro* experiments, rapamycin (200 nm) was administered 0.5 h before OGD or 4 h after OGD/R. In addition, bafilomycin A1 (baf A1) was dissolved in DMSO at a stock concentration of 0.5 mg/ml. In *in vitro* experiments, baf A1 was injected intracerebroventricularly at 120 ng per rat 2 h before the animals were killed. In the *in vitro* experiments, 200 nm baf A1 was administered 2 h before the cells were harvested. The doses were chosen according to previous reports (Sarkar et al., 2009; Zhang et al., 2009; H. Li et al., 2014).

### Blood-brain barrier permeability assay

Blood-brain barrier integrity was assessed by FITC-dextran permeability assay. Briefly, FITC-dextran (40 kDa) was injected intravenously at 21 h after MCAO/R. The mice were killed 3 h following tracer infusions, perfused, and fixed with paraformaldehyde. Brains were removed and cut into 10-µm slices. Coronal brain sections were stained with CD31 antibody to visualize the cerebrovascular system. Fluorescence images were captured, and the fluorescence intensity of tracers was calculated using ImageJ software by a blinded observer.

### Construction of expression plasmids

The coding region of mouse Ykt6 cDNA was subcloned into the pcDNA3.1 + N-eGFP expression vector. The GFP-LC3 expression vector was from Cell Biolabs (CBA-401).

### Construction of siRNAs

To knock down Sec22b expression in cultured neurons, specific siRNAs against Sec22b were obtained from GenScript. To improve the knock-down efficiency, the interference efficiency of three different siRNAs was tested. Sec22b siRNA target sequences: (I) GGAATTTGACGAGCAGCAT; (II) CCCTAAGAAGTTGGCCTTT; (III) GCATTGGATTCAAAGGCTA. Subsequent experiments and the AAV9-Sec22b shRNA-eGFP design used Sec22b-siRNA III, the most efficient siRNA.

### Transfection

hCMEC/D3 cells and neurons were transfected with expression vectors using Lipofectamine 3000 Transfection Reagent (Invitrogen, L3000-015) according to the manufacturer's instructions. At 48 h after transfection, cells were harvested to detect transfection efficiency or exposed to the indicated stimulus.

### Stereotaxic adeno-associated virus (AAV) injection

The Sec22b shRNA AAV (AAV9-Sec22b shRNA-eGFP), Ykt6 overexpression AAV (AAV9-Ykt6-eGFP) and the corresponding control AAV, with the neuron-specific promoter hSyn, were purchased from Genechem. Stereotaxic injections were made at AP: + 0.3 mm, −0.8 mm, and −1.9 mm; ML: 2.5 mm; DV: −2 mm from bregma. Mice recovered for three weeks after virus injection before experiments to allow sufficient expression of the transgene.

### Transmission electron microscopy (TEM)

TEM was performed as previously described (H. Li et al., 2013). Five ultrathin sections per rat were examined and photographed using an FEI Tecnai G2 Spirit electron microscope (FEI Tecnai).

### Measurement of mitochondrial membrane potential

The fluorescent, lipophilic and cationic probe JC-1 (C2006, Beyotime) was employed to measure the mitochondrial membrane potential of cultured neurons and BMVECs according to the manufacturer's directions. The mitochondrial membrane potential was calculated as the fluorescence ratio of red (i.e., aggregates) to green (i.e., monomers; K. Chen et al., 2009; Zhu et al., 2009).

### LysoTracker Red staining

LysoTracker Red staining was performed on cultured BMVECs according to the manufacturer's directions (C1046, Beyotime).

### Neurobehavioral assessment

Neurobehavioral studies, rotarod tests, foot fault tests, and adhesive removal tests were performed by blinded observers at the indicated time points as described previously (Shi et al., 2016; T. Yang et al., 2018; Dai et al., 2020; R. Wang et al., 2020).

### Measurements of infarct volume

Acute-stage cerebral infarction was assessed by triphenyl tetrazolium chloride (TTC) staining as described previously (Z. Wang et al., 2017). Infarct volumes were measured by a blinded observer using ImageJ software on TTC-stained sections. Infarct volumes were corrected for brain edema by reporting the volume of the contralateral hemisphere minus the noninfarcted volume of the ipsilateral hemisphere.

### Western blot and immunofluorescence analyses

Ischemic penumbra or peri-ischemic brain tissue was used for western blot and immunofluorescence analysis. The ischemic penumbra of MCAO/R model in mice was determined according to the method described in Ashwal and colleagues (Zhang et al., 2014). Western blot and immunofluorescence analyses were performed as described previously (H. Li et al., 2013).

### Antibodies

Antibodies against cleaved caspase-3 (9661), LC3 (2775), and Atg7 (2631) were purchased from Cell Signaling Technology. Sec22b antibody (14776-1-AP) was purchased from Proteintech. Ykt6 antibody (ab236583), NeuN antibody (ab104224), and GFAP antibody (ab134436) were from Abcam. CD31 antibody (AF3628) was purchased from R&D Systems. Antibodies against β-tubulin (sc-5274), normal rabbit IgG (sc-2027), and horseradish peroxidase-conjugated secondary antibodies were purchased from Santa Cruz Biotechnology. Alexa Fluor-488 donkey anti-rabbit IgG antibody (A21206), Alexa Fluor-555 donkey anti-mouse IgG antibody (A31570), and Alexa Fluor-555 donkey anti-goat IgG antibody (A21432) were purchased from Invitrogen.

### Experimental design and statistical analysis

Sample sizes for animal studies were determined by power calculations based on pilot studies (power 80%, α 0.05). GraphPad Prism 7.00 was used for all statistical analyses. D'Agostino–Pearson's K2 test was used to assess the normality of the data. Data are presented as the mean ± SD for parametric distributions and the median with interquartile range for nonparametric tests. One-way ANOVA and two-way ANOVA were used for parametric data analysis. Differences in means across multiple groups over time were analyzed using two-way repeated-measures ANOVA. The Kruskal–Wallis test, Mann–Whitney test, and χ^2^ tests were used to analyze nonparametric data. The log-rank (Mautel–Cox) test was used to analyze Kaplan–Meier survival curves. *p* < 0.05 was considered statistically significant. Exact *p* values are reported in the figure legends unless *p* < 0.0001. A detailed statistical table is shown in Extended Data Figure 1-1.

## Results

### Dual effects of pharmacological autophagy induction on acute cerebral I/R injury in a rat MCAO/R model

**Figure 1.**
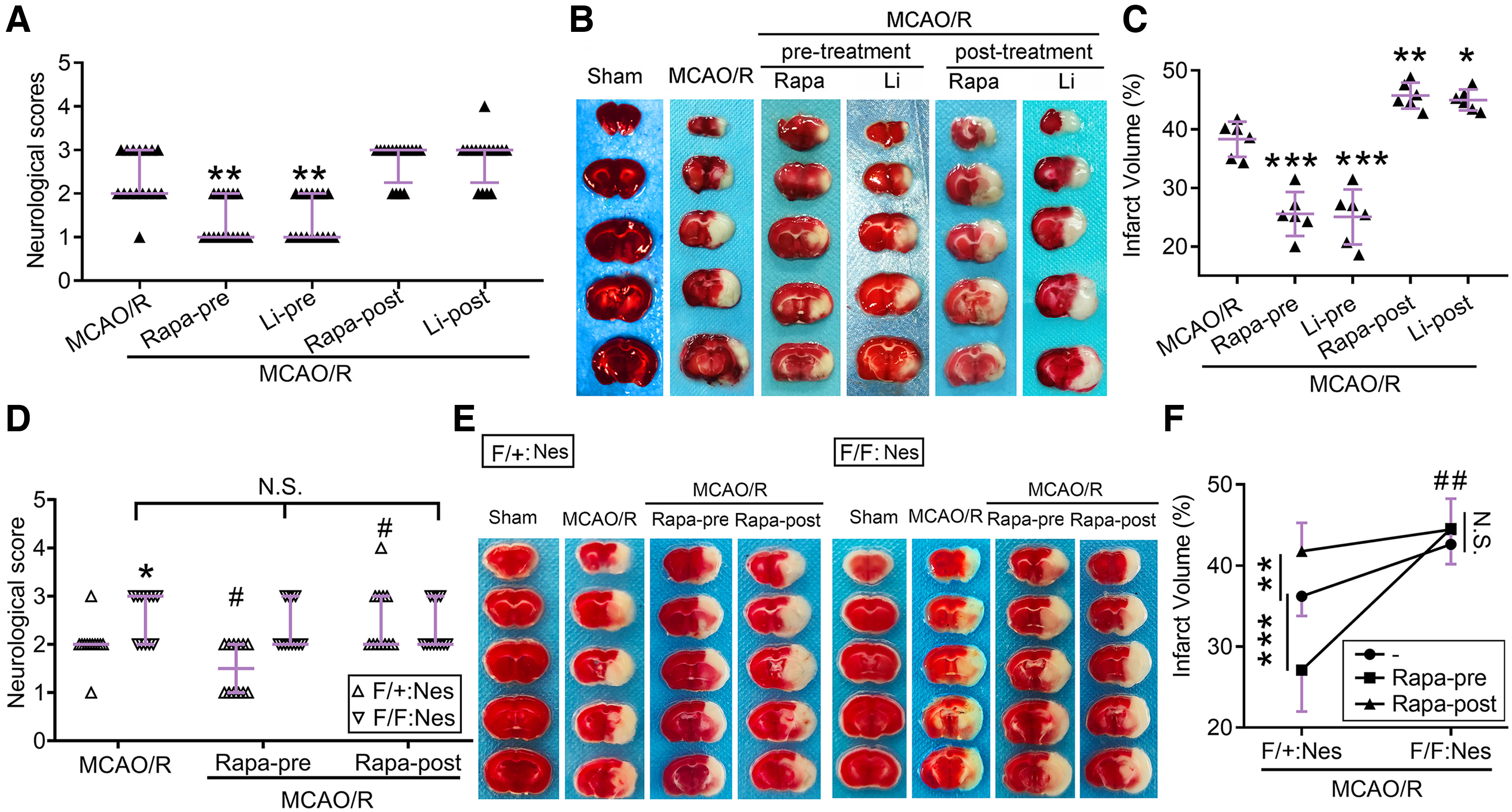
Effects of autophagy inducer treatments on the outcome of the murine MCAO/R model. ***A***, Neurologic scoring of rats 2 d after MCAO/R. Data are presented as the median with interquartile range. **p = 0.0022 for MCAO/R + Rapa-pre and **p = 0.0045 for MCAO/R + Li-pre versus MCAO/R group by Kruskal–Wallis test, *n* = 16. A detailed statistical table is shown in Extended Data Figure 1-1. ***B***, Representative TTC-stained coronal sections of rats 2 d after MCAO/R. ***C***, Statistical analysis of the infarct volume shown in ***B***. Data are presented as the mean ± SD. ***p < 0.0001, **p = 0.0045, *p = 0.012 versus MCAO/R group (*F*_(4,25)_ = 58.08, *p* < 0.0001, one-way ANOVA). *N* = 6 mice per group. ***D***, Neurobehavioral scores of mice 2 d after MCAO/R. Data are presented as the median with interquartile range. *p = 0.025, #*p* = 0.0425 versus F/+:Nes + MCAO/R group, N.S.: not significant (*F*_(1,66)_ = 11.30, *p* = 0.0013, two-way ANOVA). *N* = 12 mice per group. The phenotypes of Atg7^flox/flox^; Nestin-Cre mice and cerebral blood flow monitoring during MCAO modeling are shown in Extended Data Figures 1-2, 1-3. ***E***, Representative TTC-stained coronal sections of mice 2 d after MCAO/R. ***F***, Statistical analysis of the infarct volume shown in ***E***. Data are presented as the mean ± SD. ***p < 0.0001, **p = 0.0084, ^##^*p* = 0.0031 for F/+:Nes group versus F/F:Nes group, N.S.: not significant (*F*_(2,47)_ = 16.76, *p* < 0.0001, two-way ANOVA). *N* = 9 mice per group. F/+:Nes: Atg7^flox/+^; Nestin-Cre; F/F:Nes: Atg7^flox/flox^; Nestin-Cre.

In view of the contradiction between the prestroke and poststroke treatment results of pharmacological autophagy modulators (Shi et al., 2012), we treated the MCAO/R rat model with the pharmacological autophagy inducers rapamycin and lithium carbonate before and after treatment. At 2 d after reperfusion, compared with the MCAO/R group, the two pretreatment groups showed improved neurologic function, while posttreatment did not exhibit a significant effect (Fig. 1*A*). TTC staining also displayed the benefit of rapamycin and lithium carbonate pretreatment on MCAO/R-induced infarction, while posttreatment led to more severe infarction (Fig. 1*B*,*C*).

10.1523/JNEUROSCI.2030-21.2022.f1-1 Extended Data Figure 1-1 Detailed statistical table. Download Figure 1-1, DOCX file.

**Figure 2.**
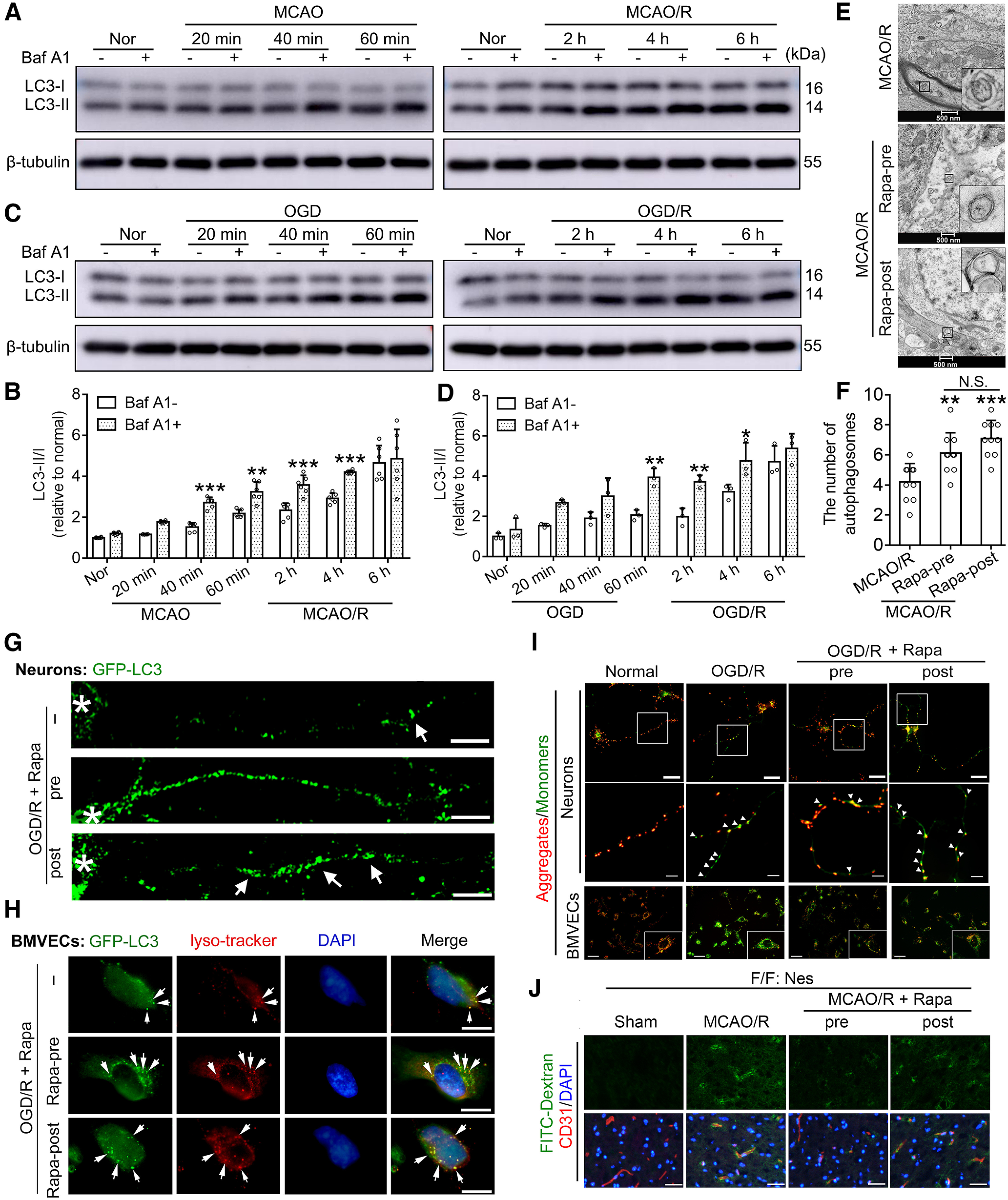
Autophagic flux determination in neurons during I/R. ***A***, Western blot analysis of LC3 in penumbra tissue of mice at the indicated time points after ischemia or after reperfusion. ***B***, The LC3-II/I ratio was calculated. Data are presented as the mean ± SD. ***p = 0.0008 for baf A1– versus baf A1+ in the MCAO 40-min group, **p = 0.0034 for baf A1– versus baf A1+ in the MCAO 60-min group, ***p = 0.0004 for baf A1– versus baf A1+ in the MCAO/R 2 h group, ***p = 0.0003 for baf A1– versus baf A1+ in the MCAO/R 4 h group (*F*_(1,70)_ = 56.44, *p* < 0.0001, two-way ANOVA). *N* = 6 mice per group. ***C***, Western blot analysis of LC3 in cultured neurons at the indicated time points after OGD or reoxygenation. ***D***, The LC3-II/I ratio was calculated. Data are presented as the mean ± SD. **p = 0.0013 for baf A1– versus baf A1+ in the 60-min OGD group, **p = 0.0027 for baf A1– versus baf A1+ in the 2-h OGD/R group, *p = 0.0107 for baf A1– versus baf A1+ in the 4-h MCAO/R OGD/R group (*F*_(1,28)_ = 53.10, *p* < 0.0001, two-way ANOVA). *N* = 3 independent replicates. ***E***, TEM of autophagosome structures in neurons of MCAO/R rats 6 h after reperfusion. ***F***, The number of autophagosomes was quantified. Data are presented as the mean ± SD. **p = 0.0048 for MCAO/R versus MCAO/R+ Rapa-pre group, ***p < 0.0001 for MCAO/R versus MCAO/R + Rapa-post group, N.S.: not significant (*F*_(2,27)_ = 13.50, *p* < 0.0001, one-way ANOVA). *N* = 10 random fields per group. ***G***, Fluorescence images of cultured neurons transfected with GFP-LC3 at 6 h after OGD/R. The asterisk shows the nucleus. The arrow indicates autophagosome accumulation. Scale bar: 20 μm. ***H***, LysoTracker Red staining of cultured BMVECs transfected with GFP-LC3 at 6 h after OGD/R. Arrows indicate double labeling with GFP-LC3 and LysoTracker Red, defined as autolysosomes. Scale bar: 10 μm. ***I***, Measurement of mitochondrial membrane potential in cultured neurons and BMVECs at 6 h after OGD/R. Scale bar: 10 μm. The arrow indicates depolarized mitochondria in the neurite structure. Original images of JC-1 and the fluorescence ratio of red (i.e., aggregates) to green (i.e., monomers) are shown in Extended Data Figure 2-1. ***J***, Representative images of FITC-dextrans (40 kDa, green) and CD31 immunofluorescence staining (red) at 24 h after MCAO/R (scale bar: 50 μm). Corresponding original images and quantification of the relative fluorescence intensity of FITC-dextran are shown in Extended Data Figure 2-2. Original images of Western blottings for this figure are shown in Extended Data Figure 2-3.

10.1523/JNEUROSCI.2030-21.2022.f1-2 Extended Data Figure 1-2 The phenotypes of Atg7flox/flox; Nestin-Cre mice. A, Reverse transcription and polymerase chain reaction analysis of genomic DNA extracted from WT, Atg7flox/+, Atg7flox/flox, and Nestin-Cre mouse tails. The amplified fragments derived from WT and mutant alleles are indicated. B, Morphology of WT, Atg7flox/+; Nestin-Cre (F/+: Nes) and Atg7flox/flox; Nestin-Cre (F/F: Nes) mice. C, Breeding genotypes. *p = 0.0282 by χ2 test. D, Brain homogenates from mice at P70 were immunoblotted with antibodies against Atg7 and LC3. The data shown are representative of three separate experiments. E, Kaplan–Meier survival curves of WT, F/+:NES and F/F:NES mice over 70 d. ***p < 0.0001. F, Body weight. ***p < 0.0001 (F(2,33) = 104.6, p < 0.0001, two-way ANOVA). N = 12 mice per genotype. G, Neurobehavioral scores at P70. N.S.: not significant (F(2,45) = 0.5000, p = 0.6099, one-way ANOVA). N = 16 mice per genotype. Download Figure 1-2, TIF file.

**Figure 3.**
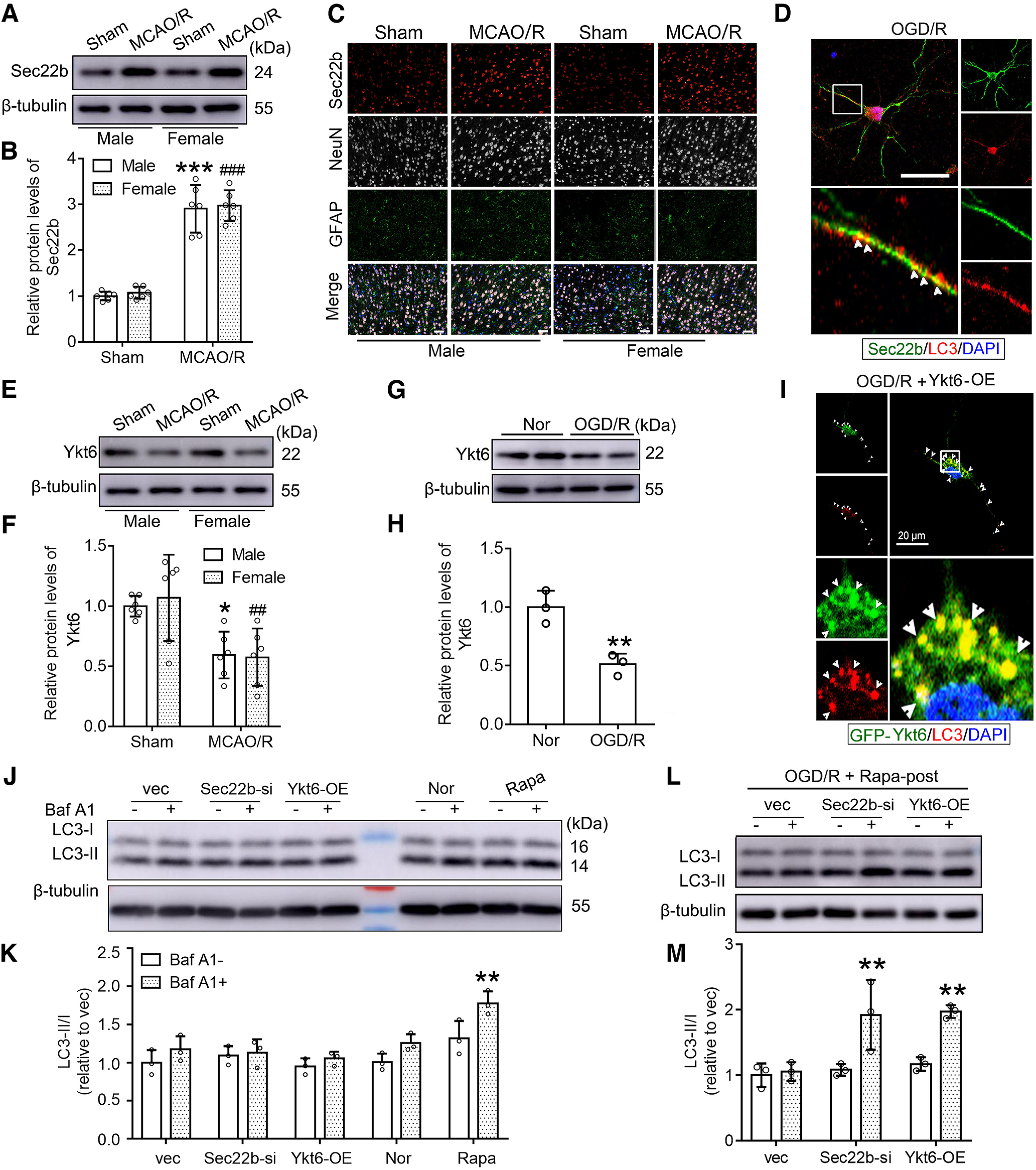
Sec22b and Ykt6 play key roles in autophagosome retrograde trafficking in neurons exposed to I/R. ***A***, Western blot analysis of Sec22b in penumbra tissue of male and female mice 6 h after MCAO/R. ***B***, The relative protein level of Sec22b was quantified. Data are presented as the mean ± SD. ***p < 0.0001 for sham versus MCAO/R in males; ###*p* < 0.0001 for sham versus MCAO/R in females (*F*_(1,20)_ = 0.2836, *p* = 0.6002, one-way ANOVA). *N* = 6 mice per group. ***C***, Immunofluorescence analysis was performed in the penumbra tissue of mice at 6 h after MCAO/R. Sec22b antibody (red), NeuN antibody (white) and GFAP antibody (green) were used, and nuclei were fluorescently labeled with DAPI (blue). Scale bar: 50 μm. ***D***, Double immunofluorescence analysis was performed with antibodies against Sec22b (green) and LC3 (red) in neurons at 6 h after OGD/R. Nuclei were fluorescently labeled with DAPI (blue). Representative images of reproducible triplicate experiments. Arrows indicate the colocalization of Sec22b and LC3 in neurites. Scale bar: 20 μm. ***E***, Western blot analysis of Ykt6 in penumbra tissue of male and female mice 6 h after MCAO/R. ***F***, The relative protein level of Ykt6 was quantified. Data are presented as the mean ± SD. *p = 0.0165 for sham versus MCAO/R in males; ##*p* = 0.0039 for sham versus MCAO/R in females (*F*_(1,20)_ = 0.06,209, *p* = 0.8058, one-way ANOVA). *N* = 6 mice per group. ***G***, Western blot assay of Ykt6 in cultured neurons 6 h after OGD/R. ***H***, The relative protein level of Ykt6 was quantified. Data are presented as the mean ± SD. **p = 0.0073 for Nor versus OGD/R group by unpaired *t* test. *N* = 3 independent replicates. ***I***, Immunofluorescence analysis was performed with an antibody against LC3 (red) in neurons overexpressing GFP-Ykt6 at 6 h after OGD/R. Nuclei were fluorescently labeled with DAPI (blue). Representative images of reproducible triplicate experiments. Arrows indicate the colocalization of GFP-Ykt6 and LC3 in the proximal neurite. Scale bar: 20 μm. ***J***, Western blotting was performed to detect the response of Sec22b knock-down and Ykt6 overexpression to baf A1 in cultured neurons under non-OGD conditions. Rapamycin treatment was used as a positive control. ***K***, The LC3-II/I ratio was calculated. Data are presented as the mean ± SD. **p = 0.0062 for baf A1– versus baf+ in the Rapa group (*F*_(1,20)_ = 13.97, *p* = 0.0013, two-way ANOVA). *N* = 3 independent replicates. ***L***, Western blot analysis of LC3 in neurons exposed to the indicated treatments 6 h after OGD/R. ***M***, The LC3-II/LC3-I ratio was calculated. **p = 0.0039 for baf A1– versus baf A1+ in Sec22b-si group; Data are presented as the mean ± SD. **p = 0.0054 for baf A1– versus baf A1+ in Ykt6-OE group (*F*_(1,12)_ = 23.73, *p* = 0.0004, two-way ANOVA). *N* = 3 independent replicates. Original images of Western blottings for this figure are shown in Extended Data Figure 3-1.

10.1523/JNEUROSCI.2030-21.2022.f1-3 Extended Data Figure 1-3 Cerebral blood flow was monitored by a laser speckle imaging system. A, Representative laser speckle images of mice before surgery (baseline), during ischemia, and 10 min after reperfusion. B, Quantitative analyses of cerebral blood flow changes showing successful ischemia and reperfusion. N.S.: not significant (F(2,45) = 0.2332, p = 0.7930, two-way ANOVA). N = 6 mice per group. Download Figure 1-3, TIF file.

### Phenotype of CNS-specific Atg7-deficient mice

To examine whether rapamycin and lithium carbonate exert effects via autophagy in this MCAO/R model, we obtained mice deficient for Atg7 specifically in the central nervous system (Atg7^flox/flox^; Nestin-Cre) and their littermate controls (Atg7^flox/+^; Nestin-Cre). The results of RT–PCR used for genotyping are shown in Extended Data Figure 1-2*A*. Although Atg7^flox/flox^; Nestin-Cre mice seemed normal at birth (Extended Data Fig. 1-2*B*), Atg7^flox/flox^; Nestin-Cre mice were born at a lower frequency than the expected Mendelian frequency (Extended Data Fig. 1-2*C*). The loss of both Atg7 and LC3-II was observed at postnatal day (P)70 (Extended Data Fig. 1-2*D*). In addition, the survival rate of the homozygous mice diminished markedly after birth and remained constant between P60 and P70 (Extended Data Fig. 1-2*E*). Atg7^flox/flox^; Nestin-Cre mice had lower body weights than heterozygous mice (Extended Data Fig. 1-2*F*). The low birth frequency and survival rate of Atg7f^lox/flox^; Nestin-Cre mice suggested that endogenous autophagy in the central nervous system plays a crucial role in development. Notably, based on the five-point method (T. Yang et al., 2018), Atg7^flox/flox^; Nestin-Cre mice had no apparent neurobehavioral defect up to 10 weeks after birth (Extended Data Fig. 1-2*G*).

### Autophagy is critical for rapamycin pretreatment-induced neuroprotection in a mouse MCAO/R model

During MCAO modeling, a laser speckle imaging system was used to monitor cerebral blood flow in mice (Extended Data Fig. 1-3). All mice were subjected to the same degree of ischemia and achieved the same extent of reperfusion. Compared with heterozygous mice, Atg7^flox/flox^; Nestin-Cre mice showed more severe behavioral deficits and infarction at 2 d after MCAO/R (Fig. 1*D–F*), suggesting that endogenous autophagy was a protective mechanism against I/R injury. Consistent with the results in rats (Fig. 1*A–C*), preischemia and postreperfusion rapamycin treatment separately rescued and enhanced MCAO/R-induced behavioral deficits and infarctions in heterozygous mice (Fig. 1*D–F*). However, rapamycin treatment before ischemia or after reperfusion did not cause significant changes in neurobehavioral abilities or infarct volume 2 d after MCAO/R in Atg7^flox/flox^ Nestin-Cre mice (Fig. 1*D–F*).

### Special neurite structure: a challenge to autophagic flux in neurons during I/R

Accumulating evidence has advanced the critical role of intact autophagic flux in the pro-survival function of autophagy, especially in terminally differentiated and nondividing neurons (Komatsu et al., 2006). As a V-ATPase inhibitor, baf A1 can block the fusion of autophagosomes and lysosomes and was used to detect autophagic flux (Mauvezin and Neufeld, 2015; Klionsky et al., 2016). Given the dynamic nature of autophagy and stroke pathology, we found that autophagy levels were upregulated in penumbra brain tissue or cultured neurons ∼40 min after ischemia, and the autophagic flux was intact. In addition, the autophagic flux level was also upregulated in penumbral tissue or cultured neurons 2 h after reperfusion compared with the normal group, while it lasted up to 4 h after reperfusion, but the autophagic flux was incomplete at 6 h after reperfusion (Fig. 2*A–D*). TEM of autophagosome structures in neurons of MCAO/R rats showed that pretreatment and posttreatment of rapamycin significantly upregulated the number of autophagosomes without a difference (Fig. 2*E*,*F*). There was a uniform and dispersed arrangement of autophagosomes in the neuronal neurite structure in the rapamycin pretreatment group, suggesting that there may be autophagosome queue transport (Fig. 2*E*). However, autophagosome accumulation in neurite terminals in the rapamycin posttreatment group suggested that there may be an obstacle in autophagosome retrograde trafficking.

10.1523/JNEUROSCI.2030-21.2022.f2-1 Extended Data Figure 2-1 Original images and quantitative analyses for Figure 2I. A, Original images of JC-1. B, The fluorescence ratio of red (i.e., aggregates) to green (i.e., monomers) in cultured neurons and BMVECs was quantified. **p = 0.0025, ***p = 0.0001 versus normal group, #p = 0.0491 for neurons and #p = 0.0174 for BMVECs versus OGD/R group, N.S.: no significant difference for neurons and &p = 0.0420 for BMVECs versus OGD/R group (F(1,16) = 13.55, p = 0.0020, two-way ANOVA). N = 3 independent replicates. Download Figure 2-1, TIF file.

10.1523/JNEUROSCI.2030-21.2022.f2-2 Extended Data Figure 2-2 Original images and quantitative analyses for Figure 2*J*. ***A***, Original images of FITC-dextrans (40 kD, green) and CD31 immunofluorescence staining (red). ***B***, Quantization of the relative fluorescence intensity of FITC-dextran shown in ***A***. Sham group was normalized to a fold value of 1. ***p < 0.0001 for MCAO/R group versus sham group, ###*p* < 0.0001 for MCAO/R + Rapa-pre group versus MCAO/R group, ##*p* = 0.0024 for MCAO/R + Rapa-post group versus MCAO/R group, &&*p* = 0.0028 for MCAO/R + Rapa-post group versus MCAO/R + Rapa-pre group (*F*_(3,20)_ = 63.54, *p* < 0.0001, one-way ANOVA). *N* = 6 independent replicates. Download Figure 2-2, TIF file.

10.1523/JNEUROSCI.2030-21.2022.f2-3 Extended Data Figure 2-3 Full original images of Western blotting assays for Figure 2. Download Figure 2-3, TIF file.

In cultured neurons transfected with GFP-LC3, an obstacle to autophagosome retrograde trafficking characterized by autophagosome accumulation in distal neurites was also detected in the posttreatment group (Fig. 2*G*). Considering the special neurite structure in neurons, we performed LysoTracker Red staining of cultured BMVECs (hCMEC/D3 cells) transfected with GFP-LC3. Double labeling with GFP-LC3 and LysoTracker Red was used to define autolysosomes (Son et al., 2012). The micrographs demonstrated that both pretreatment and posttreatment of rapamycin promoted GFP-LC3 patch formation and the co-localization of GFP-LC3 and LysoTracker in BMVECs (Fig. 2*H*). All the results suggested that the special neurite structure highlighted the importance of autophagosome transport for intact autophagic flux in neurons exposed to I/R.

Because autophagy is an important mechanism of clearing damaged mitochondria (W. Li et al., 2021), we further investigated the effects of rapamycin treatment on mitochondrial function in cultured neurons and BMVECs exposed to OGD/R. At 6 h after OGD/R, the mitochondrial membrane potential of neurons decreased, which could be inhibited by rapamycin pretreatment but not by rapamycin posttreatment (Fig. 2*I*; Extended Data Fig. 2-1). In BMVECs, both rapamycin pretreatment and posttreatment reversed OGD/R-induced mitochondrial depolarization. To test the direct effect of rapamycin on blood-brain barrier integrity independent of neuronal autophagy induction, FITC-dextran permeability assay was performed in the MCAO/R model of neuron-specific autophagy-deficient (Atg7^flox/flox^; Nestin-Cre) mice. The fluorescence signal of FITC-dextran could hardly be detected in the brain tissues of the sham group, indicating that neuron-specific autophagy deficiency did not cause blood-brain barrier injury. At 24 h after MCAO/R, 40 kDa dextran extravasation was detected in the mouse brain parenchyma, which was significantly improved by both pretreatment and posttreatment with rapamycin. Notably, rapamycin posttreatment improved blood-brain barrier integrity significantly less than rapamycin pretreatment (Fig. 2*J*; Extended Data Fig. 2-2).

### Sec22b and Ykt6 are involved in axonal autophagosome retrograde trafficking in neurons exposed to I/R

Previous reports have shown that Sec22b participates in autophagosome formation and mediates secretory autophagy (Nair et al., 2011; Daste et al., 2015). In this study, we found that Sec22b was significantly increased in the mouse penumbra tissue 6 h after MCAO/R modeling, which did not show a sex difference (Fig. 3*A*,*B*). Immunofluorescence co-staining of Sec22b and NeuN/GFAP showed that Sec22b was mainly expressed and changed in neurons, while Sec22b was almost undetectable in astrocytes (Fig. 3*C*). In addition, compared with the OGD group, the OGD/R group showed more Sec22b/LC3 co-localizations in the neurites of cultured neurons (Fig. 3*D*).

10.1523/JNEUROSCI.2030-21.2022.f3-1 Extended Data Figure 3-1 Full original images of Western blotting assays for Figure 3. Download Figure 3-1, TIF file.

It has been reported that in yeast cells, Ykt6p (Ykt6 homologous protein) is specifically highly expressed in the part missing Sec22p (Sec22b homologous protein) and makes up for Sec22p's involvement in intracellular transport, suggesting that there may be a connection between Sec22b expression and Ykt6 expression (Liu and Barlowe, 2002). Ykt6 has also been reported to be distributed in axons of neurons and participate in the transport of intracellular vesicles from axons to cell bodies (Hasegawa et al., 2004; Tai et al., 2004). Compared with the sham group, MCAO/R induced a significant decrease in Ykt6 in the mouse penumbra tissue, which did not show a sex difference (Fig. 3*E*,*F*). Because there is no commercial antibody suitable for the immunofluorescence assay of Ykt6 when we performed the work, the changes in Ykt6 in neurons were validated by western blot assay of cultured neurons. (Fig. 3*G*,*H*). Compared with the normal group, OGD/R induced a significant decrease in Ykt6 in cultured neurons. In addition, a GFP-Ykt6 expression plasmid was transfected into neurons to enhance Ykt6 expression and visualize Ykt6. The results showed that in neurons exposed to OGD/R, Ykt6/LC3 co-location was more prominent in the proximal neurite and cell soma than in the axon (Fig. 3*I*).

Based on the results shown above, we hypothesized that I/R led to changes in the protein levels of Sec22b and Ykt6, which may be responsible for the obstacle in axonal autophagosome retrograde trafficking in neurons. To test this hypothesis, we examined the effects of downregulation of Sec22b and upregulation of Ykt6 on autophagic flux in neurons, and rapamycin treatment was used as a positive control (Fig. 3*J*). The results showed that compared with the normal group, rapamycin treatment significantly upregulated LC3-II/I ratio, and baf A1 further upregulated LC3-II/I ratio, suggesting that rapamycin treatment induced autophagy in neurons with an intact autophagic flux. Compared with the vector group, Sec22b knock-down and Ykt6 overexpression did not significantly affect the LC3-II/I ratio in neurons with or without baf A1 treatment (Fig. 3*K*). These results suggested that Sec22b knock-down and Ykt6 overexpression have no significant effect on autophagy and autophagic flux in normal cultured neurons. In addition, we found that administration of baf A1 could significantly increase LC3-II in the OGD/R + rapamycin posttreatment + Ykt6 overexpression group and OGD/R + rapamycin posttreatment + Sec22b knock-down group but not in the OGD/R + rapamycin posttreatment group (Fig. 3*L*,*M*), suggesting that Ykt6 overexpression and Sec22b knock-down rescued autophagic flux damage induced by OGD/R in neurons.

### Sec22b knock-down and Ykt6 overexpression rescue cerebral I/R injury in mice

**Figure 4.**
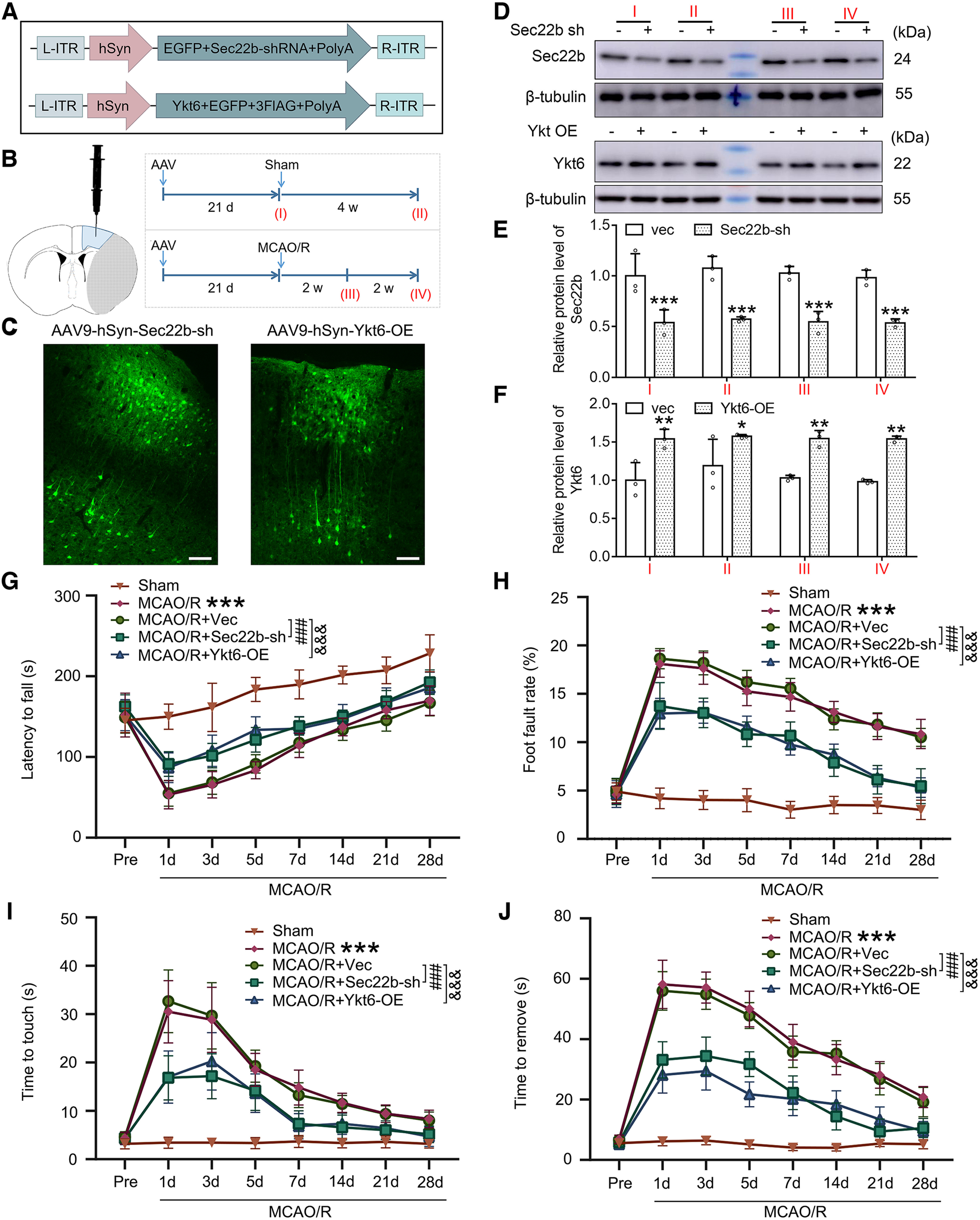
Sec22b knock-down and Ykt6 overexpression alleviate long-term sensorimotor deficits after MCAO/R. ***A***, Schematic diagram showing the experimental strategy for Sec22b knock-down and Ykt6 overexpression. Intervention efficiency of Sec22b knock-down and Ykt6 overexpression by AAV stereotactic injection. ***B***, AAV9-Sec22b shRNA-eGFP and AAV9-Ykt6-eGFP were stereotactically injected at AP: + 0.3 mm, −0.8 mm, and −1.9 mm; ML: 2.5 mm; DV: −2 mm from bregma. The shaded area indicates the AAV intervention areas sampled in this experiment, and the sampling procedure for this experiment is shown. ***C***, Representative micrographs of AAV9-Sec22b shRNA-eGFP and AAV9-Ykt6-eGFP transfection characteristically in neurons are shown. Scale bar: 50 μm. ***D***, Western blot analysis of Sec22b and Ykt6 at the indicated time points. ***E***, Relative protein expression of Sec22b was calculated. Data are presented as the mean ± SD. ***p = 0.0005 for I: vec - Sec22b-sh group, ***p = 0.0002 for II: vec - Sec22b-sh group, ***p = 0.0003 for III: vec - Sec22b-sh group, ***p = 0.0007 for IV: vec - Sec22b-sh group (*F*_(1,16)_ = 107.5, *p* < 0.0001, two-way ANOVA). *N* = 3 independent replicates. ***F***, The relative protein expression of Ykt6 was calculated. Data are presented as the mean ± SD. **p = 0.0029 for I: vec - Ykt6-OE group, *p = 0.0381 for II: vec - Ykt6-OE group, **p = 0.0042 for III: vec - Ykt6-OE group, **p = 0.0022 for IV: vec - Ykt6-OE group (*F*_(1,16)_ = 59.20, *p* < 0.0001, two-way ANOVA). *N* = 3 independent replicates. ***G***, Rotarod test. The time between placing the mice on the rotating drum and falling was recorded. ***H***, Foot fault test. The proportion of wrong steps of the left upper limb in all steps of mice was calculated. ***I***, ***J***, Adhesive removal test. The time for the mice to touch and remove the stickers was recorded and statistically analyzed. (***G***-***L***) Data are presented as the mean ± SD. ***p < 0.001 versus sham group, ###*p* < 0.001, &&&*p* < 0.001 by two-way ANOVA with Tukey's multiple comparisons test, *n* = 12. Original images of Western blottings for this figure are shown in Extended Data Figure 4-1.

To further evaluate the potential roles of Sec22b and Ykt6 in cerebral I/R injury, specific Sec22b knock-down and Ykt6 overexpression in penumbra neurons were performed by stereotaxic injection of AAV9 with the specific promoter hSyn in mice (Fig. 4*A*,*B*). At 21 d after AAV injection, GFP signaling was detected in neuron-like cells surrounding the injection site (Fig. 4*C*). Consistent with the fluorescence observation of GFP, AAV significantly downregulated Sec22b protein levels and upregulated Ykt6 protein levels after 21 d of injection, and the effects lasted up to four weeks under sham and MCAO/R conditions (Fig. 4*D–F*). We then assessed the effects of exogenous regulation on the sensorimotor functions of mice using the rotarod test (Fig. 4*G*), foot fault test (Fig. 4*H*), and adhesive removal test (Fig. 4*I*,*J*). MCAO mice exhibited significantly deteriorated sensorimotor impairments compared with sham mice up to 28 d after surgery. Vector intervention did not affect the sensorimotor functions of MCAO-operated mice, while both Sec22b knock-down and Ykt6 overexpression significantly alleviated MCAO/R-induced sensorimotor impairments.

10.1523/JNEUROSCI.2030-21.2022.f4-1 Extended Data Figure 4-1 Full original images of Western blotting assays for Figure 4. Download Figure 4-1, TIF file.

### Sec22b knock-down and Ykt6 overexpression rescue cerebral I/R injury in an autophagy-dependent manner

**Figure 5.**
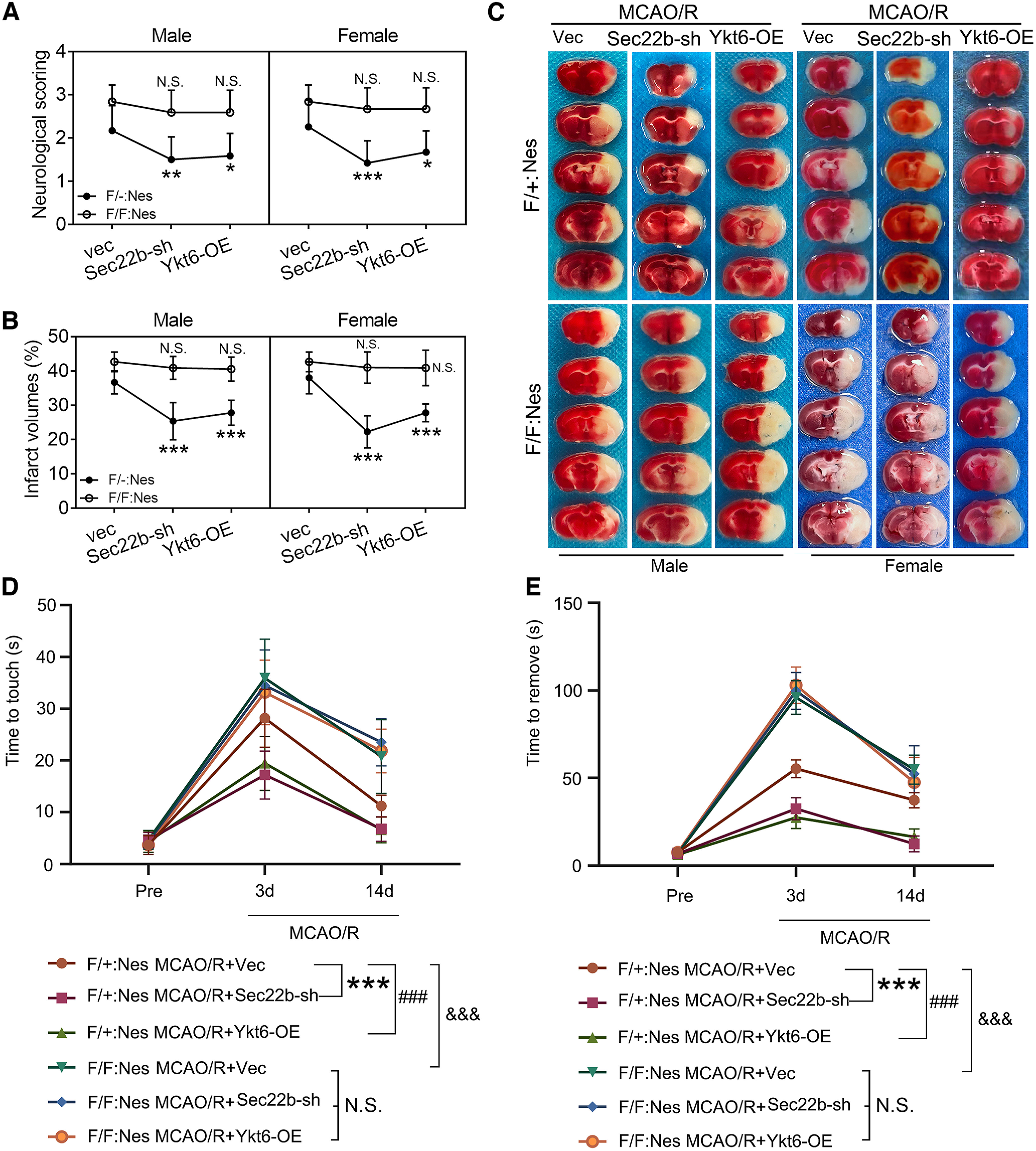
Sec22b knock-down and Ykt6 overexpression induce neuroprotection in MCAO/R mice via autophagy. ***A***, Neurologic scoring of male and female mice at 2 d after MCAO/R. Data are presented as the mean ± SD. Male: **p = 0.0032 for F/+:Nes:MCAO/R + vec versus MCAO + Sec22b-sh group; *p = 0.0111 for MCAO/R + vec versus MCAO + Ykt6-OE group; Female: ***p = 0.0002 for F/+:Nes:MCAO/R + vec versus MCAO + Sec22b-sh group; *p = 0.0111 for MCAO/R + vec versus MCAO + Ykt6-OE group. N.S.: not significant (*F*_(3,132)_ = 40.53, *p* < 0.0001, two-way ANOVA). *N* = 9 mice per group. ***B***, Statistical analysis of the infarct volume of male and female mice assessed by TTC staining at 2 d after MCAO/R. Data are presented as the mean ± SD. Male: ***p < 0.0001 for F/+:Nes:MCAO/R + vec versus MCAO + Sec22b-sh group; ***p < 0.0001 for MCAO/R + vec versus MCAO + Ykt6-OE group; female: ***p < 0.0001 for F/+:Nes:MCAO/R + vec versus MCAO + Sec22b-sh group; ***p < 0.0001 for MCAO/R + vec versus MCAO + Ykt6-OE group. N.S.: not significant (*F*_(3,96)_ = 78.80, *p* < 0.0001, two-way ANOVA). *N* = 9 mice per group. ***C***, Representative TTC staining images of male and female mice at 2 d after MCAO/R. ***D***, ***E***, Adhesive removal test. The time for the mice to touch and remove the stickers was recorded and statistically analyzed. Data are presented as the mean ± SD. ***p < 0.001, ###*p* < 0.001, &&&*p* < 0.001, N.S.: not significant by two-way ANOVA with Tukey's multiple comparisons test, *n* = 12. F/+:Nes: Atg7^flox/+^; Nestin-Cre; F/F:Nes: Atg7^flox/flox^; Nestin-Cre.

To further explore the mechanism of exogenous Sec22b knock-down and Ykt6 overexpression in improving brain injury, we further performed exogenous interventions before MCAO modeling in Atg7^flox/flox^; Nestin-Cre mice and littermate controls (Atg7^flox/+^; Nestin-Cre). We found that Sec22b knock-down and Ykt6 overexpression could significantly inhibit MCAO/R-induced behavioral dysfunction and infarction in heterozygous mice, which did not show significant sex differences and was eliminated in Atg7^flox/flox^; Nestin-Cre mice (Fig. 5), suggesting that endogenous autophagy is indispensable for Sec22b knock-down and Ykt6 overexpression-induced neuroprotection against I/R injury.

### Sec22b knock-down and Ykt6 overexpression reversed the outcome of rapamycin posttreatment in MCAO/R mice

**Figure 6.**
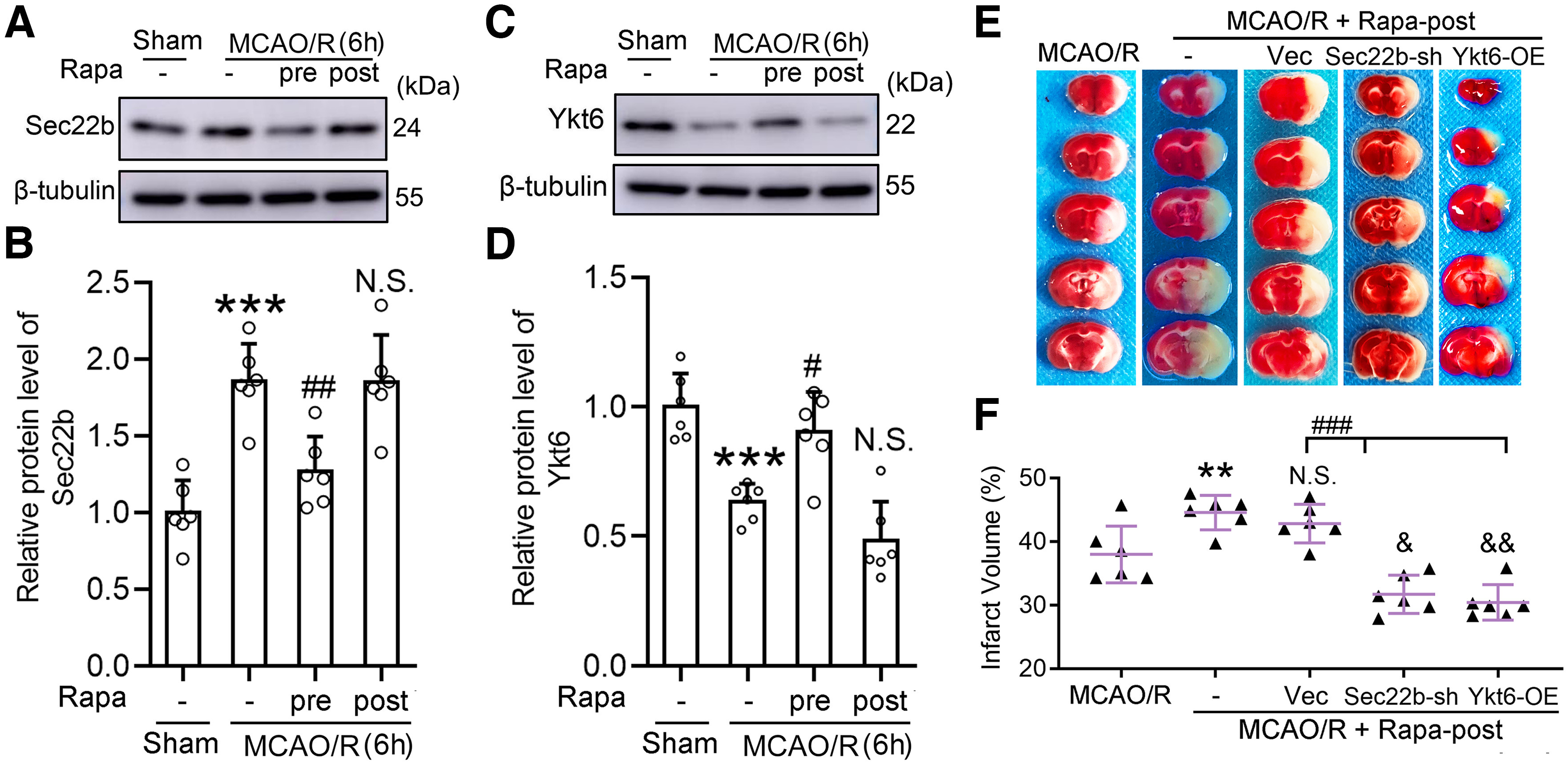
Sec22b knock-down and Ykt6 overexpression improved the outcomes of rapamycin posttreatment in MCAO/R mice. ***A***, Western blot analysis of Sec22b in the penumbra brain tissue 6 h after MCAO/R. ***B***, The relative protein level of Sec22b was quantified. Data are presented as the mean ± SD. ***p < 0.0001 for MCAO/R versus sham group, ##*p* = 0.0038 for MCAO/R + Rapa-pre versus MCAO/R group, N.S.: not significant (*F*_(3,20)_ = 17.50, *p* < 0.0001, one-way ANOVA). *N* = 6 mice per group. ***C***, Western blot analysis of Ykt6 in the penumbra brain tissue 6 h after MCAO/R. ***D***, The relative protein level of Ykt6 was quantified. Data are presented as the mean ± SD. ***p = 0.0005 for MCAO/R versus sham group, #*p* = 0.0109 for MCAO/R + Rapa-pre versus MCAO/R group, N.S.: not significant (*F*_(3,20)_ = 20.12, *p* < 0.0001, one-way ANOVA). *N* = 6 mice per group. ***E***, Representative TTC staining images of mice at 2 d after MCAO/R. ***F***, Statistical analysis of infarct volume. Data are presented as the mean ± SD. **p = 0.0072, &*p* = 0.0162, &&*p* = 0.003 versus MCAO/R group, N.S.: not significant versus MCAO/R + Rapa-post group, ###*p* < 0.0001 (*F*_(4,25)_ = 22.73, *p* < 0.0001, one-way ANOVA). *N* = 6 mice per group. Original images of Western blottings for this figure are shown in Extended Data Figure 6-1.

As shown in Figure 2, rapamycin pretreatment induced an intact autophagic flux in neurons exposed to I/R. We tested the effects of rapamycin treatment on the protein levels of Sec22b and Ykt6 in neurons exposed to OGD/R (Fig. 6*A–D*). The results showed that rapamycin pretreatment could inhibit the MCAO/R-induced increase in Sec22b and decrease in Ykt6 but not posttreatment. In addition, both Sec22b knock-down and Ykt6 overexpression inhibited rapamycin posttreatment-induced deterioration in infarction in a mouse MCAO/R model (Fig. 6*E*,*F*).

10.1523/JNEUROSCI.2030-21.2022.f6-1 Extended Data Figure 6-1 Full original images of Western blotting assays for Figure 6. Download Figure 6-1, TIF file.

## Discussion

**Figure 7.**
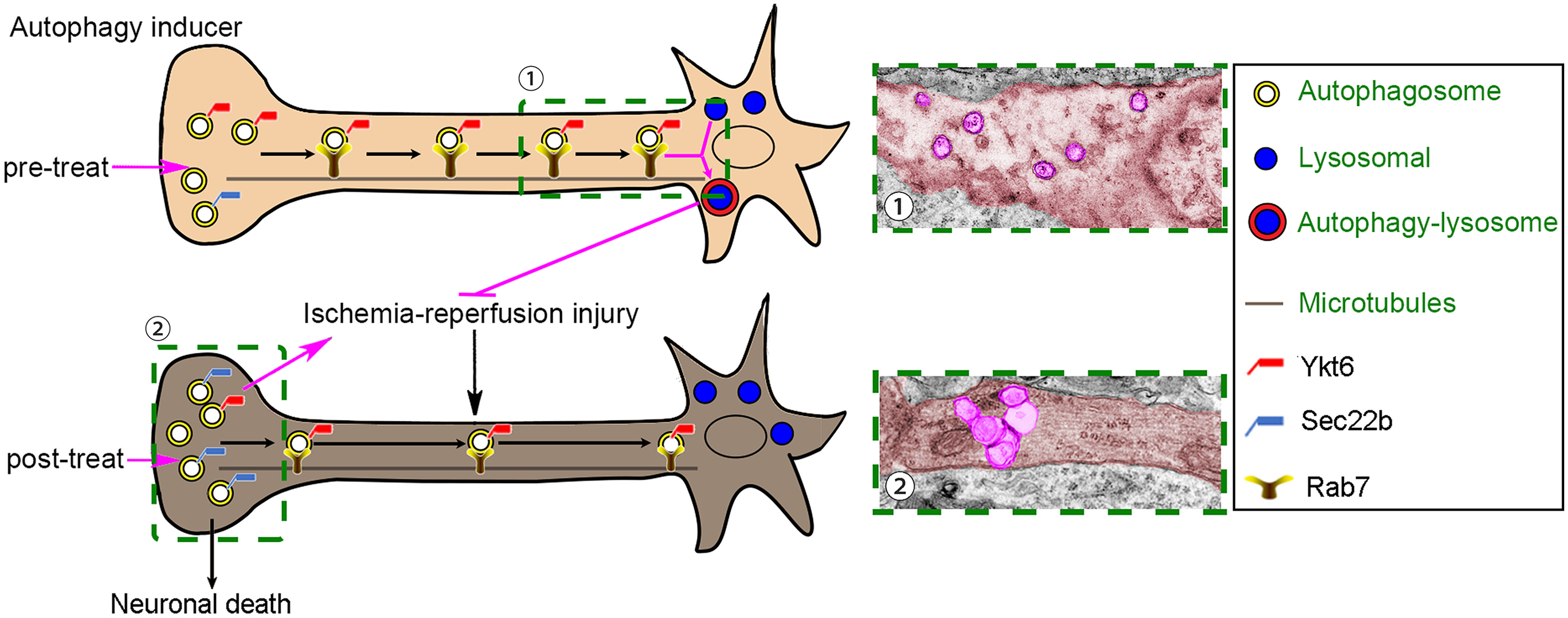
Schematic representations of the potential mechanisms of Sec22b and Ykt6 in autophagosome retrograde trafficking and autophagic flux in neurons exposed to I/R. The special neurite structure of neurons leads to unique localizations of lysosomes and autophagosomes. Lysosomes are mainly located in the cell soma, while autophagosomes are preferentially generated at the neurite tip. The balance of Sec22b and Ykt6 expression is essential for autophagosome retrograde trafficking and autophagic flux. The reperfusion-induced increase in Sec22b and decrease in Ykt6 disrupt autophagosome transport and lead to damaged autophagic flux. Autophagy inducer pretreatment could rescue reperfusion-induced disordered expression of Sec22b and Ykt6, maintain autophagic flux, and finally alleviate I/R-induced neuronal injury. In contrast, once reperfusion-induced autophagic flux damage occurs, autophagy inducer posttreatment leads to excessive autophagosome accumulation in neurite terminals and even neuronal injury.

The effect of pharmacological activation of autophagy on the outcome of cerebral ischemia remains controversial. The special neurite structure of the neurons highlights the importance of autophagosome retrograde trafficking for an intact autophagic flux (Fig. 7). In this study, we found that autophagy was activated in the penumbra, and reperfusion may result in the destruction of autophagic flux, which may be the reason for the failure of targeted autophagy induction to improve the prognosis of patients with ischemic stroke. In addition, we verified the key role of Sec22b and Ykt6 in the autophagic flux of neurons. The imbalance of Sec22b and Ykt6 caused by cerebral I/R disrupts axonal autophagosome retrograde transport, which leads to incomplete autophagic flux and aggravates neuronal damage. Autophagy inducer pretreatment could rescue reperfusion-induced disordered expression of Sec22b and Ykt6, maintain autophagic flux and mitochondrial function, and finally inhibit I/R-induced neuronal injury. Sec22b knock-down and Ykt6 overexpression could switch the outcome of rapamycin posttreatment on infarction from deterioration to improvement, suggesting that modest regulation of Sec22b and Ykt6 could extend the therapeutic window of pharmacological autophagy induction for neuroprotection in cerebral ischemia.

In mammals, Ykt6 is specifically expressed in neurons and is mainly distributed in axons, participating in the transport of intracellular vesicles from axons to cell somas (Smith and Leach, 2003; Hasegawa et al., 2004). Similar to Ykt6, Sec22b is involved in the formation of autophagosomes and is widely involved in the transport of intracellular proteins and vesicles (Daste et al., 2015). In addition, recent studies have shown that Sec22b is involved in autophagy secretion, which is an unconventional secretion mechanism termed “autosecretion” (Deretic et al., 2012). Sheng's laboratory reported that late endosome-loaded dynein-snapin complexes drive autophagosome retrograde transport in axons on fusion of autophagosomes with late endosome into amphisomes (Cheng et al., 2015). Whether autophagic flux damage induced by reperfusion leads to neuronal autophagy secretion and whether Sec22b and Ykt6 are involved in the fusion of autophagosomes with late endosomes need further investigation.

Consistent with previous reports (Liu and Barlowe, 2002), we also found that there may be a connection between Sec22b and Ykt6 expression. Furthermore, miR-134 negatively regulates the expression of Ykt6 (Ruiz-Martinez et al., 2016). Under the condition of cerebral ischemia, miR-134 inhibitors play a neuroprotective role (W. Chi et al., 2014). Excitotoxic glutamate insult, a common pathologic phenomenon following I/R, has been shown to contribute to a disruption of autophagic flux in cultured hippocampal neurons (Kulbe et al., 2014). The study also reported that the application of rapamycin and trehalose (an mTOR-independent autophagy inducer) at an earlier time point before glutamate insult could enhance autophagy and protect cells from excitotoxic death. All these reports suggest that excitotoxic glutamate insult and miR-134 may regulate Sec22b and Ykt6 expression in neurons exposed to I/R.

In this study, we chose to employ Nestin-Cre to obtain mice deficient for Atg7 specifically in the central nervous system (Atg7^flox/flox^; Nestin-Cre). It has been reported that Atg7^flox/flox^; Nestin-Cre mice showed behavioral defects, including abnormal limb-clasping reflexes and a reduction in coordinated movement and growth retardation, and died within 28 weeks of birth (Komatsu et al., 2006). Consistently, in this study, Atg7^flox/flox^; Nestin-Cre mice had a lower mean body weight than heterozygote mice (Extended Data Fig. 1-2*F*). Notably, based on the five-point method, a method suitable for the evaluation of neurologic dysfunction after MCAO (T. Yang et al., 2018), Atg7^flox/flox^; Nestin-Cre mice had no apparent neurobehavioral defects up to 10 weeks after birth (Extended Data Fig. 1-2*G*). In addition, we found that the survival rate of the homozygous mice diminished markedly after birth and remained constant between P60 and P70 (Extended Data Fig. 1-2*E*). Thus, Atg7^flox/flox^; Nestin-Cre mice and their littermate controls (Atg7^flox/+^; Nestin-Cre) were used at 10 weeks after birth (weighing ∼20 g) in this study. To compensate for the specific defect of Atg7^flox/flox^; Nestin-Cre mice in neuron research, we conducted a series of verification experiments on primary neurons (Figs. 2, 3). Furthermore, other alternatives, such as CAMKII-Cre, have been employed for neuronal-specific gene manipulation (Kim et al., 2018). Nixon RA reported that TRGL6 mice achieved selective and stable expression of dual fluorescently labeled LC3 (mRFP-EGFP-LC3) in neurons, which facilitated studies of autophagy-lysosomal pathway dynamics *in vivo* (Lee et al., 2019). Transgenic mice are expected to show more intuitively the autophagic flux of neurons during brain I/R. We will take advantage of a more accurate model to study neuronal autophagy in further studies.

It has been reported that prestroke rapamycin treatment could inhibit blood-brain barrier permeability in a murine ischemia model (O.Z. Chi et al., 2017). As shown in Figure 2, both rapamycin pretreatment and posttreatment reversed OGD/R-induced mitochondrial depolarization in cultured BMVECs and MCAO/R-induced blood-brain barrier damage in neuron-specific autophagy-deficient mice. The results suggested that the protective effect of rapamycin treatment on brain injury may be due not only to rapamycin's effects on neuronal autophagy but also to endothelial protection. However, in the *in vitro* OGD/R model, rapamycin pretreatment and posttreatment improved BMVEC mitochondrial membrane potential without significant differences, while in the *in vivo* MCAO/R model, rapamycin posttreatment improved blood-brain barrier damage significantly less than rapamycin pretreatment. The neurovascular unit is a coordinated and interactional system of neurons, astrocytes, and microvessels in the brain. Complex *in vivo* environments, such as neuronal damage caused by cerebral I/R and rapamycin posttreatment, may disrupt the improvement of rapamycin posttreatment on blood-brain barrier damage. In addition to BMVECs and neurons, a protective effect of AMPK-dependent autophagy in astrocytes exposed to OGD has been observed (Gabryel et al., 2014), while impairment of autophagic flux or autophagic-lysosomal dysfunction in astrocytes could lead to cell death (Huenchuguala et al., 2014). Microglial autophagy primarily participates in the regulation of cytokine production in the brain (Song et al., 2015), and cerebral ischemia- induced microglia autophagy is closely associated with ischemic neural inflammation and injury (Z. Yang et al., 2015). The discovery of the cell specificity of autophagy may offer a new avenue for further dissection of the therapeutic potential of autophagy in ischemic stroke.

In this study, AAV intervention was performed to achieve Sec22b knock-down and Ykt6 overexpression in a mouse MCAO/R model, which is not convenient for clinical transformation. As shown in Figure 6, rapamycin pretreatment significantly inhibited MCAO/R-mediated upregulation of Sec22b and downregulation of Ykt6, suggesting that autophagy induction under physiological conditions before stroke may have the potential to indirectly regulate the expression of autophagosome transporters and promote the integrity of autophagic flux. Studies have shown that many commonly accepted lifespan-extending and healthspan-extending habits, such as exercise and calorie restriction, share the ability to activate autophagy (López-Otín et al., 2016). Whether daily exercise and calorie restriction regulate Sec22b and Ykt6 expression deserves further investigation.

This study involved pretreatment and posttreatment interventions of rapamycin and lithium carbonate, with a difference in execution time between pretreatment and posttreatment of ∼5.5 h. Studies have shown that rapamycin pretreatment can improve the rupture of the blood–brain barrier in the early stage of MCAO/R in rats. However, rapamycin pretreatment has no significant effect on blood–brain barrier permeability after MCAO/R in diabetic rats, suggesting that different body states may lead to different responses to autophagy induction (O.Z. Chi et al., 2017). In this study, the effects of rapamycin and lithium carbonate pretreatment and posttreatment on MCAO/R brain injury in mice were different, at least partly because of the different states of penumbra brain tissue when autophagy induction occurred; for example, MCAO/R led to changes in Sec22b and Ykt6 expression. The pharmacokinetics of rapamycin and lithium carbonate are affected by environmental factors, including pH (Trepanier et al., 1998; Yamaguchi et al., 2019). Further analysis of the pharmacokinetics of rapamycin and lithium carbonate under cerebral I/R conditions, especially the changes in the levels of rapamycin and lithium carbonate in brain tissues, will provide more clues for improving cerebral I/R injury through autophagy induction.

Controversies concerning the role of autophagy in ischemic stroke may be because of differences in intervention time, cell specificity, or the lack of complete analysis of autophagy processes. The potential preclinical significance of this finding is that autophagy induction, when given in conjunction with autophagosome retrograde trafficking protection, may be delivered more feasibly and safely to clinical treatment.
