# Supplementary material for: Unbalanced Regulation of Sec22b and Ykt6 Blocks Autophagosome Axonal Retrograde Flux in Neuronal Ischemia–Reperfusion Injury
Source: J Neurosci. 2022 Jul 13;42(28):5641–54. doi: 10.1523/JNEUROSCI.2030-21.2022 (PMC9295843; doi:10.1523/JNEUROSCI.2030-21.2022)
Supplement: Extended Data Figure 1-1 — Detailed statistical table. Download Figure 1-1, DOCX file. [file ns-JN-RM-2030-21-s01.docx]

**Extended Data Table: Detailed statistical table**

| Figures | Description | Testing time | In vivo or in vitro | Test used | Detail value | One- or two- tailed P value? |
| --- | --- | --- | --- | --- | --- | --- |
| Figure 1A | Neurological score | MCAO/R 2 d | In vivo | Kruskal-wa  llis test | MCAO/R vs.MCAO/R Rapa-pre:Mean Diff =26.69, P=0.0022;MCAO/R vs.MCAO/R Li-pre:Mean Diff =25.16, P=0.0045 | Two-tailed |
| Figure 1C | Infarct volume (%) | MCAO/R 2 d | In vivo | One-way ANOVA  Tukey's multiple comparisons test | F (4, 25) = 58.08，P<0.0001  MCAO/R vs.MCAO/R Rapa-pre:Mean Diff=12.74,95% CI=  7.222 to 18.25,P<0.0001;MCAO/R vs.MCAO/R Li-pre:Mean Diff=13.24,95% CI=  7.722 to 18.75,P<0.0001  MCAO/R vs.MCAO/R Rapa-post:Mean Diff=-7.452,95% CI=  -12.97 to -1.936,P=0.0045;MCAO/R vs.MCAO/R Li-post:Mean Diff=-6.690,95% CI=  -12.21 to -1.174,P=0.0120 | Two-tailed |
| Figure 1D | Neurological score | MCAO/R 2 d | In vivo | Two-way ANOVA  Dunnett's multiple comparisons test | F (1, 66) = 11.30,P=0.0013  F/+:Nes + MCAO/R vs.F/+: Nes + MCAO/R Rapa-pre: Mean Diff=0.5000, 95% CI=0.01474 to 0.9853, P=0.0425  F/+:Nes + MCAO/R vs.F/+: Nes + MCAO/R Rapa-post: Mean Diff=-0.5000, 95% CI=-0.9853 to -0.01474, P=0.0425 | Two-tailed |
| Figure 1F | Infarct volume (%) | MCAO/R 2 d | In vivo | Two-way  ANOVA  Sidak's multiple comparisons test | F (2, 47) = 16.76, P<0.0001  F/+:Nes + MCAO/R vs.F/+: Nes + MCAO/R Rapa-pre: Mean Diff=9.111, 95% CI=4.809 to 13.41, P<0.0001  F/+:Nes + MCAO/R vs.F/+: Nes + MCAO/R Rapa-post: Mean Diff=-5.556, 95% CI=-9.858 to -1.254, P=0.0084 | Two-tailed |
| Figure 2B | LC3-II/I ratio | MCAO 20min, 40min,60min and MCAO/R 2h, 4h, 6h | In vivo | Two-way  ANOVA  Bonferroni's multiple comparisons test | F (1, 70) = 56.44,P<0.0001  Baf A1- vs Baf A1+ in MCAO 40min, Mean Diff=-1.191, 95% CI=-1.997 to -0.3839, ***p=0.0008; Baf A1- vs Baf A1+ in MCAO 60min ,Mean Diff=-1.065, 95% CI=-1.872 to -0.2584, **p=0.0034; Baf A1- vs Baf A1+ in MCAO/R 2h ,Mean Diff=-1.242, 95% CI=-2.049 to -0.4353,***p=0.0004; Baf A1- vs Baf A1+ in MCAO/R 4h, Mean Diff=-1.274, 95% CI=-2.081 to -0.4675,***p=0.0003. | Two-tailed |
| Figure 2D | LC3-II/I ratio | OGD  20min, 40min,60min and OGD/R 2h, 4h, 6h | In vitro | Two-way  ANOVA  Bonferroni's multiple comparisons test | F (1, 28) = 53.10,P<0.0001  Baf A1- vs Baf A1+ in OGD 60min,Mean Diff=-1.871, 95% CI=-3.134 to -0.6088, **p=0.0013; Baf A1- vs Baf A1+ in OGD/R 2h,Mean Diff=-1.756, 95% CI=-3.019 to -0.4939, **p=0.0027; Baf A1- vs Baf A1+ in MCAO/R OGD/R 4h,Mean Diff=-1.528, 95% CI=-2.790 to -0.2657, *p=0.0107 | Two-tailed |
| Figure 2F | The number of autophagosomes | MCAO/R 6h | In vivo | One-way ANOVA  Bonferroni's multiple comparisons test | F (2, 27) = 13.50,P<0.0001  MCAO/R vs MCAO/R+ Rapa-pre,Mean Diff=-1.900, 95% CI=-3.246 to -0.5543, p=0.0048; MCAO/R vs MCAO/R+ Rapa-post,Mean Diff=-2.900, 95% CI=-4.246 to -1.554, p<0.0001. |  |
| Figure 3B | Relative protein level of Sec22b | MCAO/R 6h | In vivo | Two-way ANOVA  Bonferroni's multiple comparisons test | F (1, 20) = 0.2836,P=0.6002  Sham vs. MCAO/R in male: Mean Diff=-1.904, 95% CI=-2.351 to -1.456, P<0.0001; Sham vs. MCAO/R in female: Mean Diff=-1.898, 95% CI=-2.346 to -1.451, P<0.0001 | Two-tailed |
| Figure 3F | Relative protein level of Ykt6 | MCAO/R 6h | In vivo | One-way ANOVA  Bonferroni's multiple comparisons test | F (1, 20) = 0.06209,P=0.8058  Sham vs. MCAO/R in male: Mean Diff=0.4065, 95% CI=0.07061 to 0.7424, P=0.0165; Sham vs. MCAO/R in female: Mean Diff=0.4936, 95% CI=0.1577 to 0.8295, P=0.0039 | Two-tailed |
| Figure 3G | Relative protein level of Ykt6 | OGD/R 6h | In vitro | Unpaired t test | Nor vs. OGD/R: Mean Diff = -0.4882, 95%CI=-0.7573 to -0.2190, p=0.0073 | Two-tailed |
| Figure 3K | LC3-II/I ratio | OGD/R 6h | In vitro | Two-way ANOVA  Sidak's multiple comparisons test | F (1, 12) = 23.73,P=0.0004  Sec22b-si:BafA1-vs.BafA1+ :Mean Diff =-0.8368, 95%CI=-1.393 to -0.2809, p=0.0039;Ykt6-over:BafA1-vs.BafA1+ :Mean Diff =-0.8000, 95%CI=-1.356 to -0.2441, p=0.0054 | Two-tailed |
| Figure 4C | Latency to fall (s) | MCAO/R 1d-28d | In vivo | Two-way ANOVA  Tukey's multiple comparisons test | F (4, 440) = 266.4,P<0.0001  Sham vs. MCAO/R: Mean Diff = 66.73，95% CI=60.22 to 73.23, p<0.0001;MCAO/R Vec vs. MCAO/R Sec22b-sh: Mean Diff = -24.86，95% CI=-31.37 to -18.36, p<0.0001;MCAO/R Vec vs. MCAO/R Ykt6-OE: Mean Diff = -23.44，95% CI=-29.94 to -16.93, p<0.0001 | Two-tailed |
| Figure 4D | Foot fault rate (%) | MCAO/R 1d-28d | In vivo | Two-way ANOVA  Tukey's multiple comparisons test | F (4, 440) = 940.2,P<0.0001  Sham vs. MCAO/R: Mean Diff =-9.464，95% CI=-9.966 to -8.962, p<0.0001;MCAO/R Vec vs. MCAO/R Sec22b-sh: Mean Diff = 4.451，95% CI=3.949 to 4.953, p<0.0001;MCAO/R Vec vs. MCAO/R Ykt6-OE: Mean Diff = 4.483，95% CI=3.981 to 4.985, p<0.0001 | Two-tailed |
| Figure 4E | Time to touch (s) | MCAO/R 1d-28d | In vivo | Two-way ANOVA  Tukey's multiple comparisons test | F (4, 440) = 241.1,P<0.0001  Sham vs. MCAO/R: Mean Diff =-12.42，95% CI=-13.72 to -11.12, p<0.0001;MCAO/R Vec vs. MCAO/R Sec22b-sh: Mean Diff =6.323，95% CI=35.022 to 7.624, p<0.0001;MCAO/R Vec vs. MCAO/R Ykt6-OE: Mean Diff = 5.969，95% CI=4.667 to 7.270, p<0.0001 | Two-tailed |
| Figure 4F | Time to remove (s) | MCAO/R 1d-28d | In vivo | Two-way ANOVA  Tukey's multiple comparisons test | F (4, 440) = 860.7,P<0.0001  Sham vs. MCAO/R: Mean Diff =-31.41，95% CI=-33.13 to -29.68, p<0.0001;MCAO/R Vec vs. MCAO/R Sec22b-sh: Mean Diff =15.06，95% CI=313.34 to 16.79, p<0.0001;MCAO/R Vec vs. MCAO/R Ykt6-OE: Mean Diff = 17.01，95% CI=15.29 to 18.73, p<0.0001 | Two-tailed |
| Figure 5A | Neurological score | MCAO/R 2 d | In vivo | Two-way ANOVA  Bonferroni's multiple comparisons test | F (3, 132) = 40.53,P<0.0001  Male: F/+:Nes:MCAO/R vec vs. MCAO + Sec22b-sh:Mean Diff =0.6667，95% CI=0.1975 to 1.136, p=0.0032;MCAO/R vec vs. MCAO + Ykt6-OE:Mean Diff =0.5833，95% CI=0.1141 to 1.053, p=0.0111;  Female: F/+:Nes:MCAO/R vec vs. MCAO + Sec22b-sh:Mean Diff =0.8333，95% CI=0.3641 to 1.303, p=0.0002; MCAO/R vec vs. MCAO + Ykt6-OE:Mean Diff =0.5833，95% CI=0.1141 to 1.053, p=0.0111. | Two-tailed |
| Figure 5B | Infarct volume (%) | MCAO/R 2 d | In vivo | Two-way ANOVA  Bonferroni's multiple comparisons test | F (3, 96) = 78.80,P<0.0001  Male: F/+:Nes:MCAO/R vec vs. MCAO + Sec22b-sh:Mean Diff =11.33，95% CI=7.044 to 15.62, p<0.0001;MCAO/R vec vs. MCAO + Ykt6-OE:Mean Diff =8.889，95% CI=4.600 to 13.18, p<0.0001;  Female: F/+:Nes:MCAO/R vec vs. MCAO + Sec22b-sh:Mean Diff =15.78，95% CI=11.49 to 20.07, p<0.0001; MCAO/R vec vs. MCAO + Ykt6-OE:Mean Diff =10.22，95% CI=5.933 to 14.51, p<0.0001. | Two-tailed |
| Figure 5D | Time to touch (s) | MCAO/R 1d-28d | In vivo | Two-way ANOVA  Tukey's multiple comparisons test | F (5, 198) = 50.39,P<0.0001  F/+:Nes:MCAO/R vs. MCAO + Sec22b-sh:Mean Diff =4.806，95% CI=1.834 to 7.777, p<0.0001;MCAO/R vs. MCAO + Ykt6-OE:Mean Diff =4.222，95% CI=1.251 to 7.194, p=0.0009;F/+:Nes MCAO/R vs. F/F:Nes MCAO/R:Mean Diff =-6.111，95% CI=-9.083 to -3.140, p<0.0001 | Two-tailed |
| Figure 5E | Time to remove (s) | MCAO/R 1d-28d | In vivo | Two-way ANOVA  Tukey's multiple comparisons test | F (5, 198) = 196.0,P<0.0001  F/+:Nes:MCAO/R vs. MCAO + Sec22b-sh:Mean Diff =16.14，95% CI=11.01 to 21.27, p<0.0001;MCAO/R vs. MCAO + Ykt6-OE:Mean Diff =16.67，95% CI=11.54 to 21.80, p<0.0001;F/+:Nes MCAO/R vs. F/F:Nes MCAO/R:Mean Diff =-19.19，95% CI=-24.33 to -14.06, p<0.0001 | Two-tailed |
| Figure 6B | Relative protein level of Sec22b | MCAO/R 6h | In vivo | One-way ANOVA  Bonferroni's multiple comparisons test | F (3, 20) = 17.50,P<0.0001  Sham vs. MCAO/R: Mean Diff =-0.8529，95% CI=-1.277 to -0.4292, p<0.0001;MCAO/R vs. MCAO/R Rapa-pre: Mean Diff =0.5862，95% CI=0.1625 to 1.010, p=0.0038 | Two-tailed |
| Figure 6D | Relative protein level of Ykt6 | MCAO/R 6h | In vivo | One-way ANOVA  Bonferroni's multiple comparisons test | F (3, 20) = 20.12, P<0.0001  Sham vs. MCAO/R: Mean Diff =0.3685，95% CI=0.1479 to 0.5891, p=0.0005;MCAO/R vs. MCAO/R Rapa-pre: Mean Diff =-0.2708，95% CI=-0.4914 to -0.05024, p=0.0109 | Two-tailed |
| Figure 6F | Infarct volume (%) | MCAO/R 2 d | In vivo | One-way ANOVA | F (4, 25) = 22.73, P<0.0001  MCAO/R vs.MCAO/R Rapa-post:Mean Diff =-6.262，95% CI=-11.72 to -0.8006, p=0.0211;MCAO/R vs.MCAO/R Rapa-post Sec22b-sh:Mean Diff =6.286，95% CI=0.8245 to 11.75, p=0.0205;MCAO/R vs.MCAO/R Rapa-post Ykt6-OE:Mean Diff =7.548，95% CI=2.086 to 13.01, p=0.0049;MCAO/R Rapa-post vs.MCAO/R Rapa-post Sec22b-sh:Mean Diff =12.55，95% CI=7.086 to 18.01, p<0.0001;MCAO/R Rapa-post vs.MCAO/R Rapa-post Ykt6-OE:Mean Diff =13.81，95% CI=8.348 to 19.27, p<0.0001 | Two-tailed |
