# Supplementary figures and images for: Unbalanced Regulation of Sec22b and Ykt6 Blocks Autophagosome Axonal Retrograde Flux in Neuronal Ischemia–Reperfusion Injury
Source: J Neurosci. 2022 Jul 13;42(28):5641–54. doi: 10.1523/JNEUROSCI.2030-21.2022 (PMC9295843; doi:10.1523/JNEUROSCI.2030-21.2022)

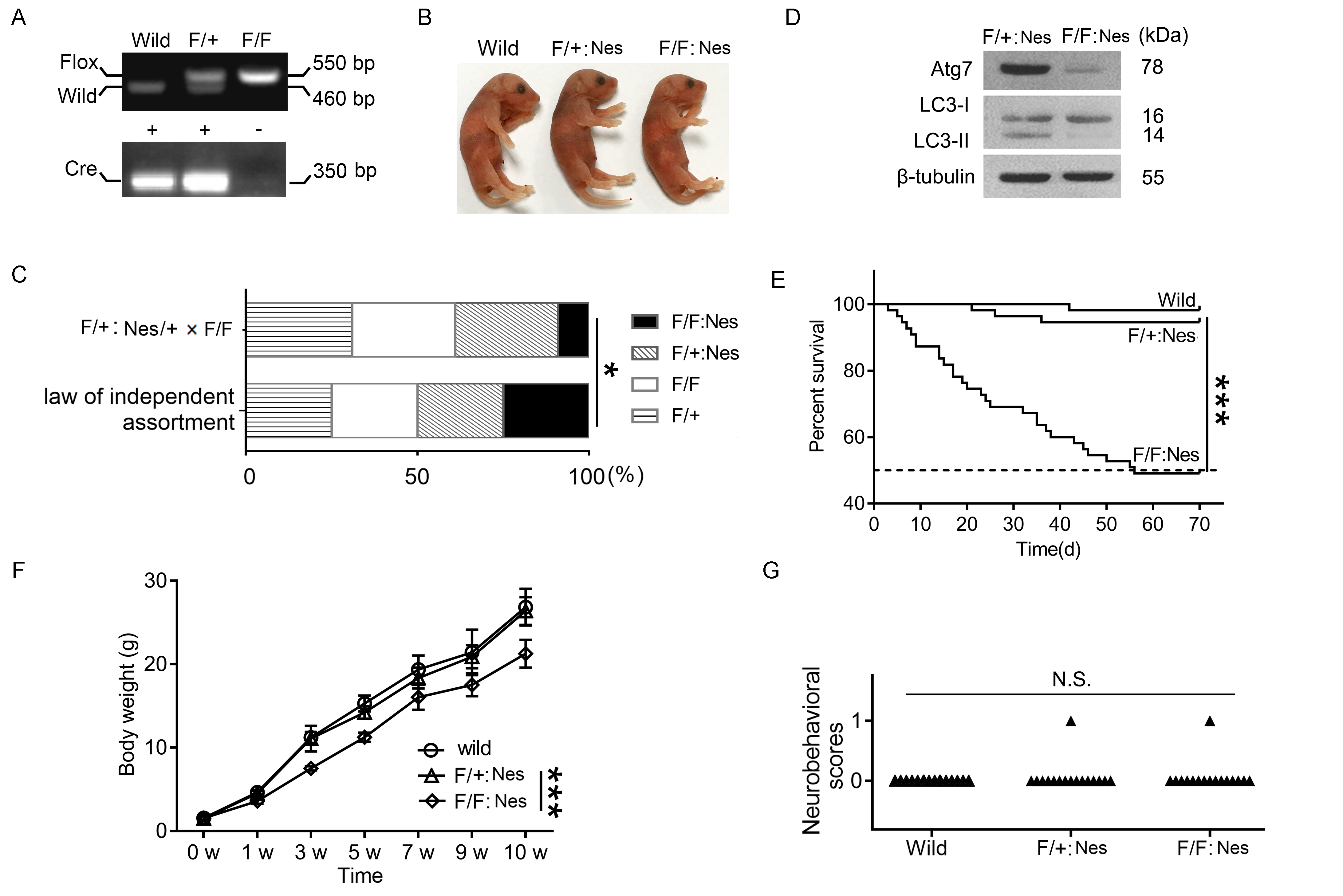

Supplement: Extended Data Figure 1-2 — The phenotypes of Atg7flox/flox; Nestin-Cre mice. A, Reverse transcription and polymerase chain reaction analysis of genomic DNA extracted from WT, Atg7flox/+, Atg7flox/flox, and Nestin-Cre mouse tails. The amplified fragments derived from WT and mutant alleles are indicated. B, Morphology of WT, Atg7flox/+; Nestin-Cre (F/+: Nes) and Atg7flox/flox; Nestin-Cre (F/F: Nes) mice. C, Breeding genotypes. *p = 0.0282 by χ2 test. D, Brain homogenates from mice at P70 were immunoblotted with antibodies against Atg7 and LC3. The data shown are representative of three separate experiments. E, Kaplan–Meier survival curves of WT, F/+:NES and F/F:NES mice over 70 d. ***p < 0.0001. F, Body weight. ***p < 0.0001 (F(2,33) = 104.6, p < 0.0001, two-way ANOVA). N = 12 mice per genotype. G, Neurobehavioral scores at P70. N.S.: not significant (F(2,45) = 0.5000, p = 0.6099, one-way ANOVA). N = 16 mice per genotype. Download Figure 1-2, TIF file. [file ns-JN-RM-2030-21-s02.tif]

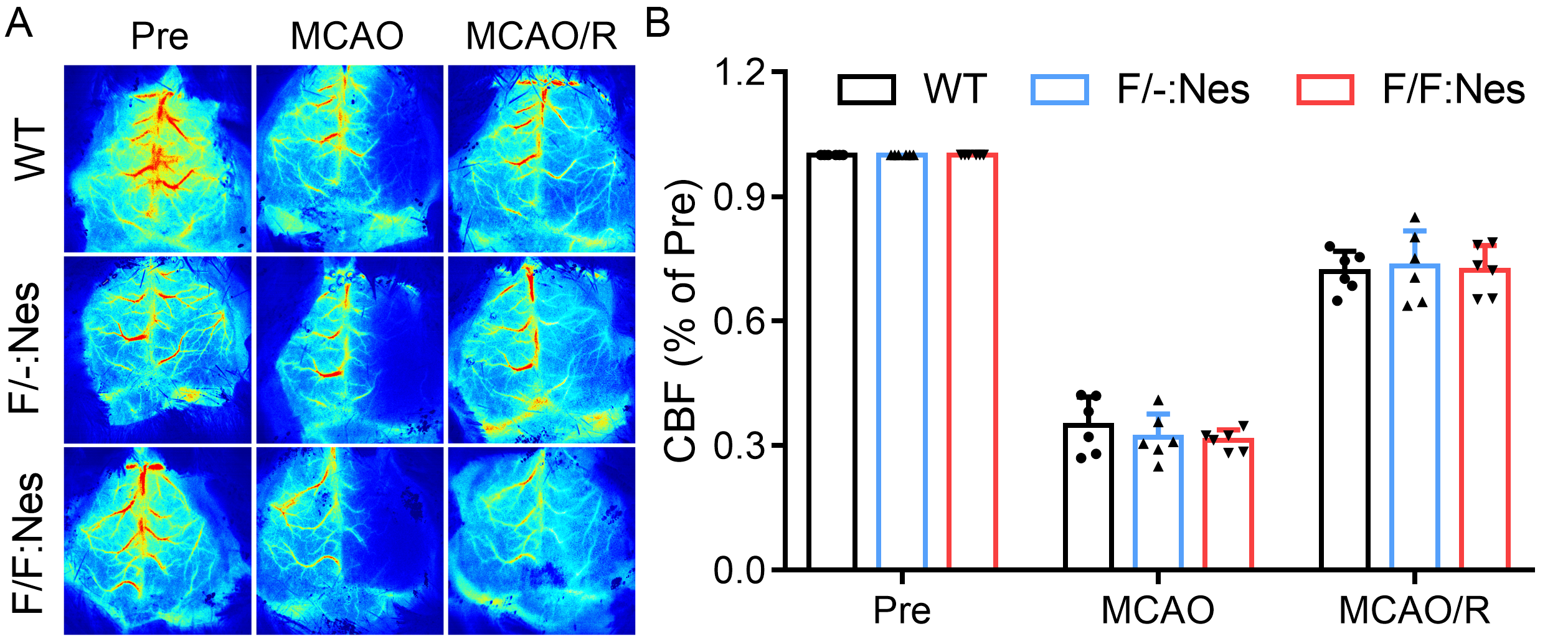

Supplement: Extended Data Figure 1-3 — Cerebral blood flow was monitored by a laser speckle imaging system. A, Representative laser speckle images of mice before surgery (baseline), during ischemia, and 10 min after reperfusion. B, Quantitative analyses of cerebral blood flow changes showing successful ischemia and reperfusion. N.S.: not significant (F(2,45) = 0.2332, p = 0.7930, two-way ANOVA). N = 6 mice per group. Download Figure 1-3, TIF file. [file ns-JN-RM-2030-21-s03.tif]

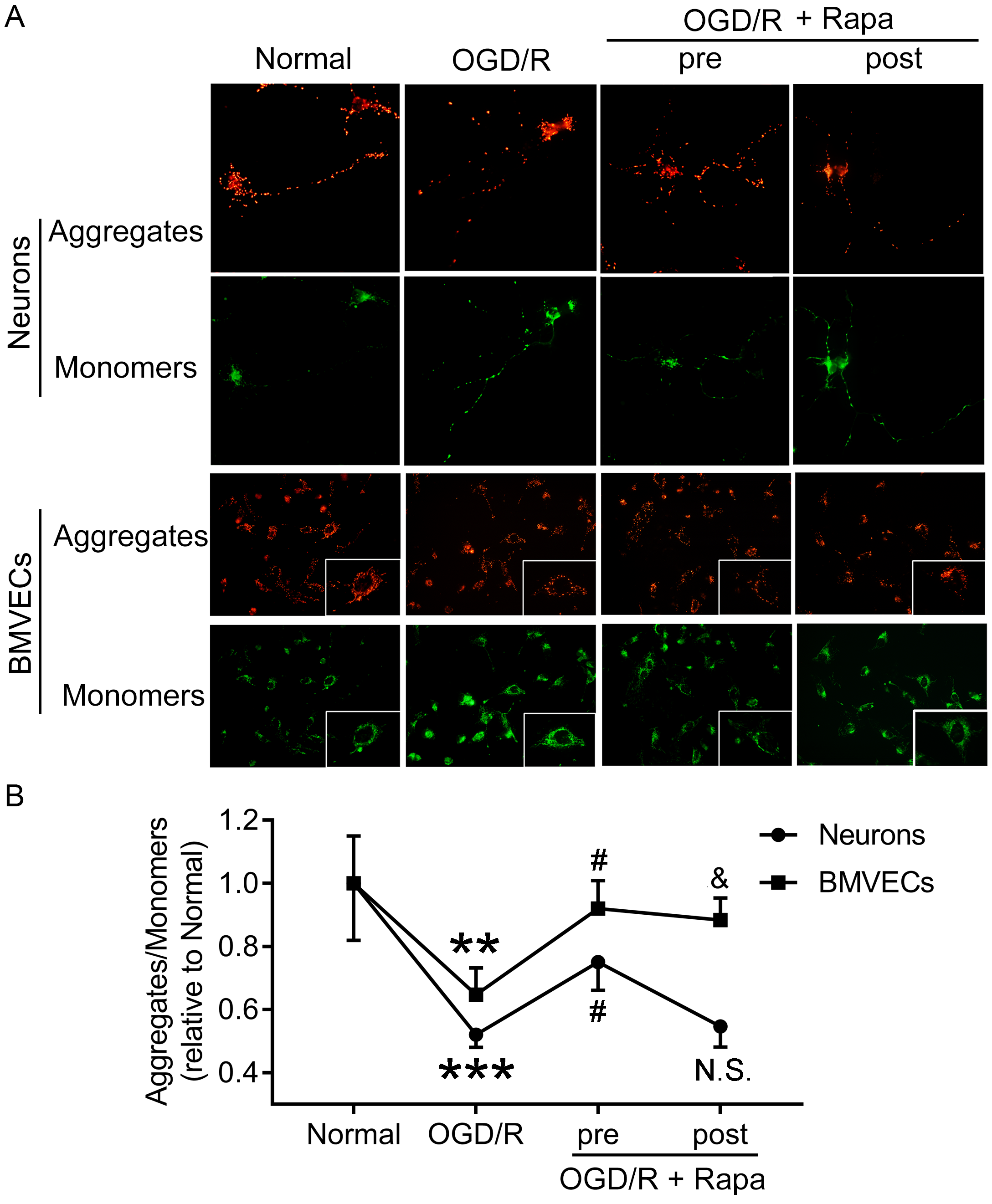

Supplement: Extended Data Figure 2-1 — Original images and quantitative analyses for Figure 2I. A, Original images of JC-1. B, The fluorescence ratio of red (i.e., aggregates) to green (i.e., monomers) in cultured neurons and BMVECs was quantified. **p = 0.0025, ***p = 0.0001 versus normal group, #p = 0.0491 for neurons and #p = 0.0174 for BMVECs versus OGD/R group, N.S.: no significant difference for neurons and &p = 0.0420 for BMVECs versus OGD/R group (F(1,16) = 13.55, p = 0.0020, two-way ANOVA). N = 3 independent replicates. Download Figure 2-1, TIF file. [file ns-JN-RM-2030-21-s04.tif]

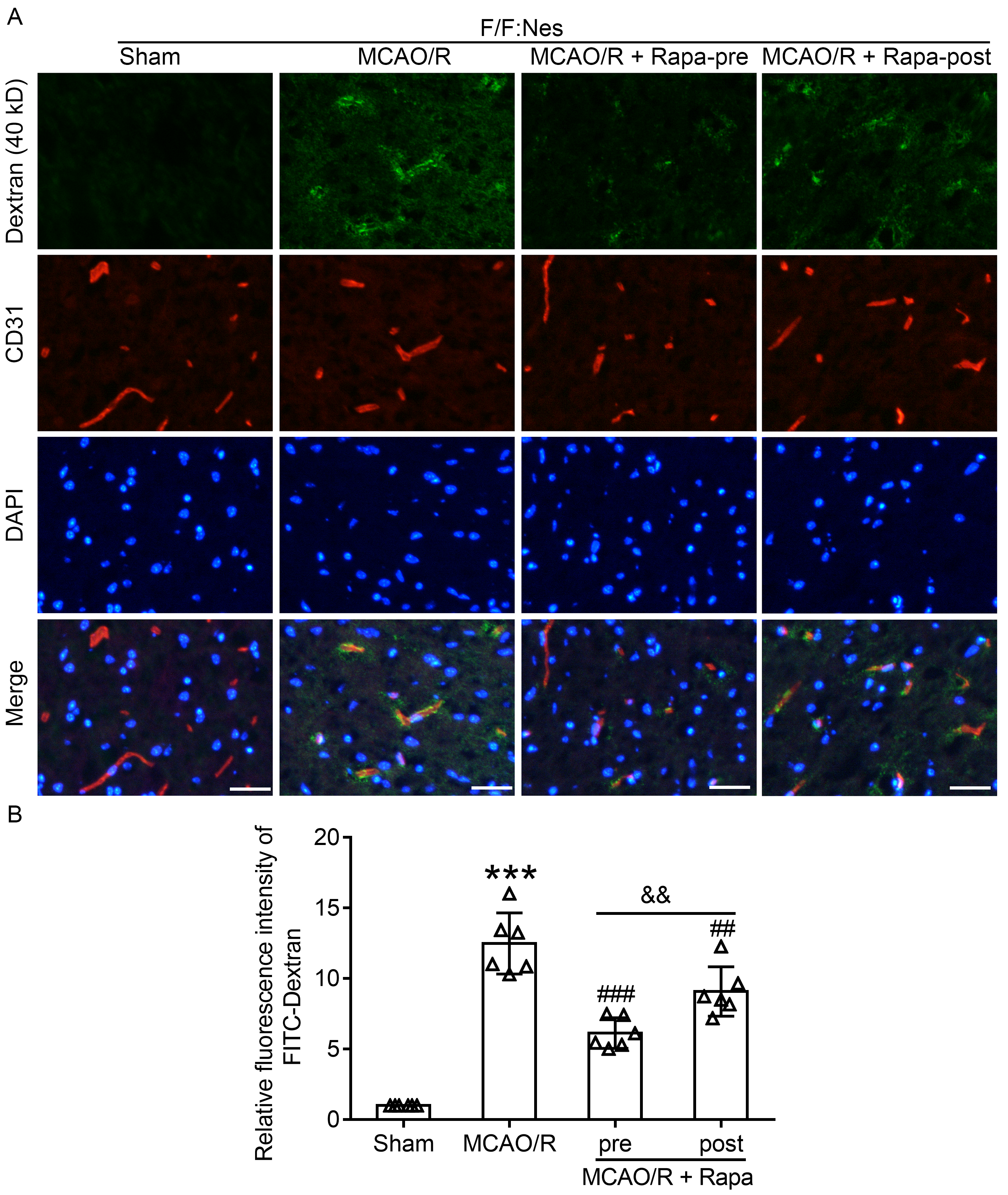

Supplement: Extended Data Figure 2-2 — Original images and quantitative analyses for Figure 2J. A, Original images of FITC-dextrans (40 kD, green) and CD31 immunofluorescence staining (red). B, Quantization of the relative fluorescence intensity of FITC-dextran shown in A. Sham group was normalized to a fold value of 1. ***p < 0.0001 for MCAO/R group versus sham group, ###p < 0.0001 for MCAO/R + Rapa-pre group versus MCAO/R group, ##p = 0.0024 for MCAO/R + Rapa-post group versus MCAO/R group, &&p = 0.0028 for MCAO/R + Rapa-post group versus MCAO/R + Rapa-pre group (F(3,20) = 63.54, p < 0.0001, one-way ANOVA). N = 6 independent replicates. Download Figure 2-2, TIF file. [file ns-JN-RM-2030-21-s05.tif]

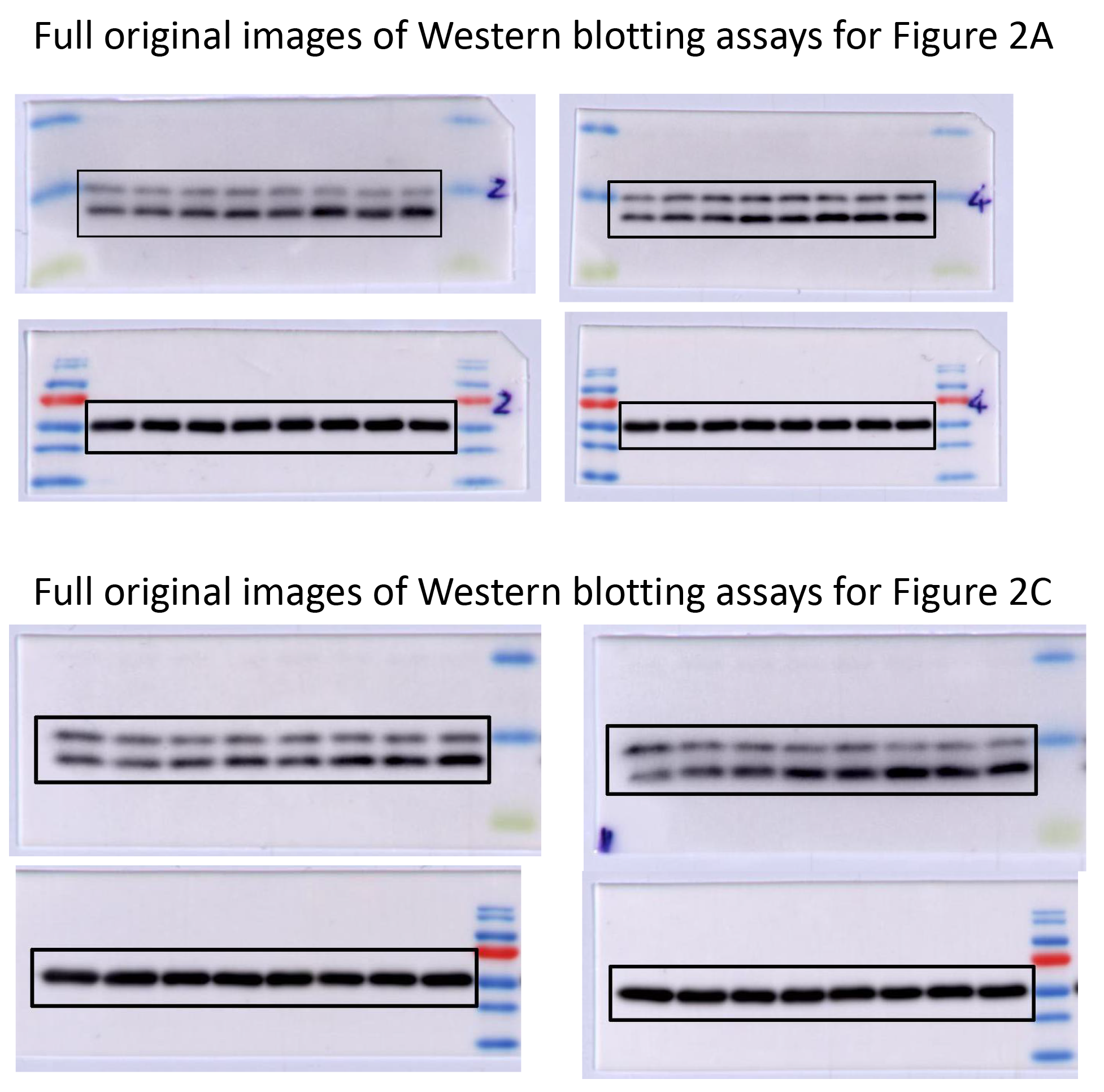

Supplement: Extended Data Figure 2-3 — Full original images of Western blotting assays for Figure 2. Download Figure 2-3, TIF file. [file ns-JN-RM-2030-21-s06.tif]

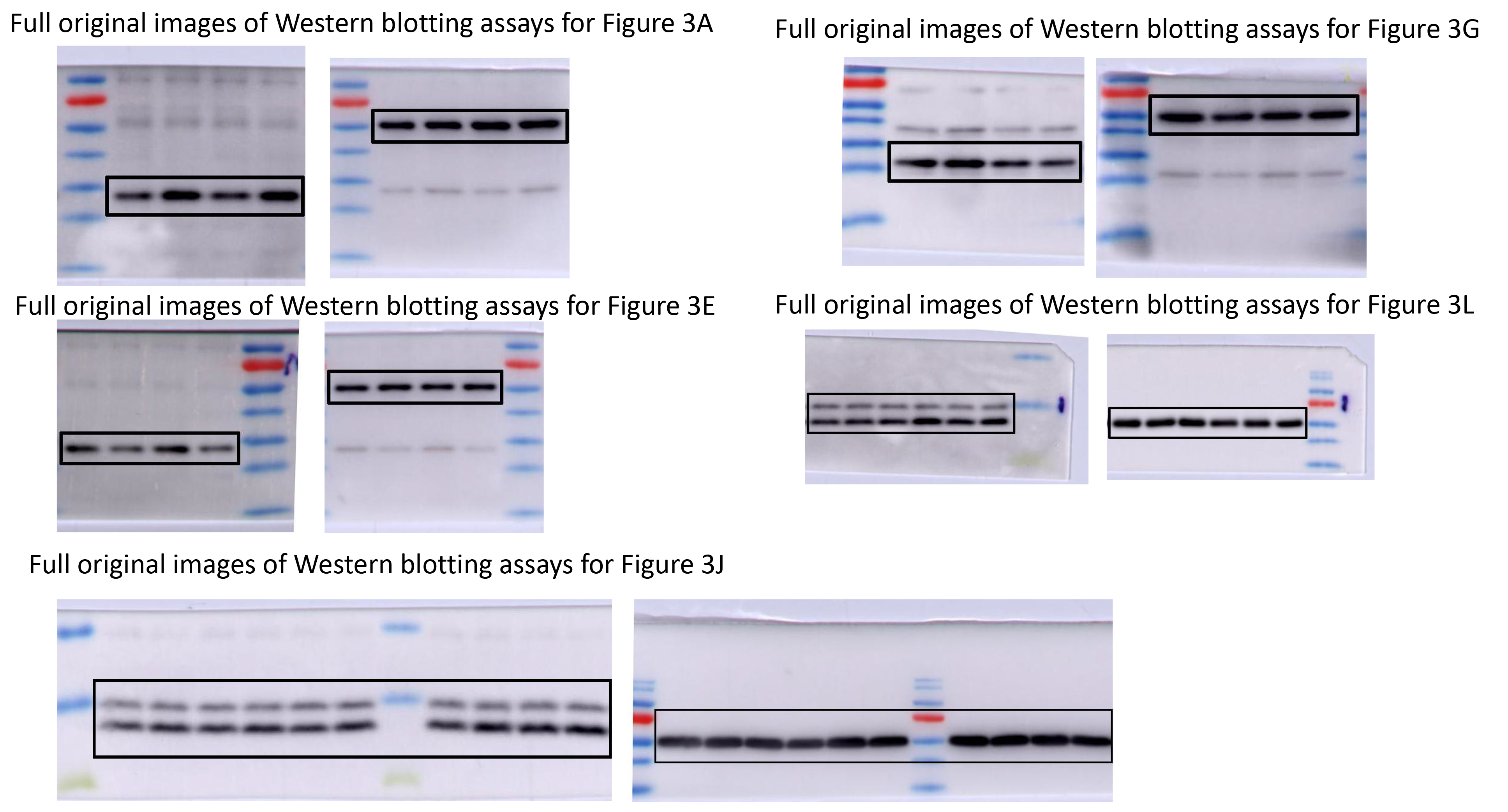

Supplement: Extended Data Figure 3-1 — Full original images of Western blotting assays for Figure 3. Download Figure 3-1, TIF file. [file ns-JN-RM-2030-21-s07.tif]

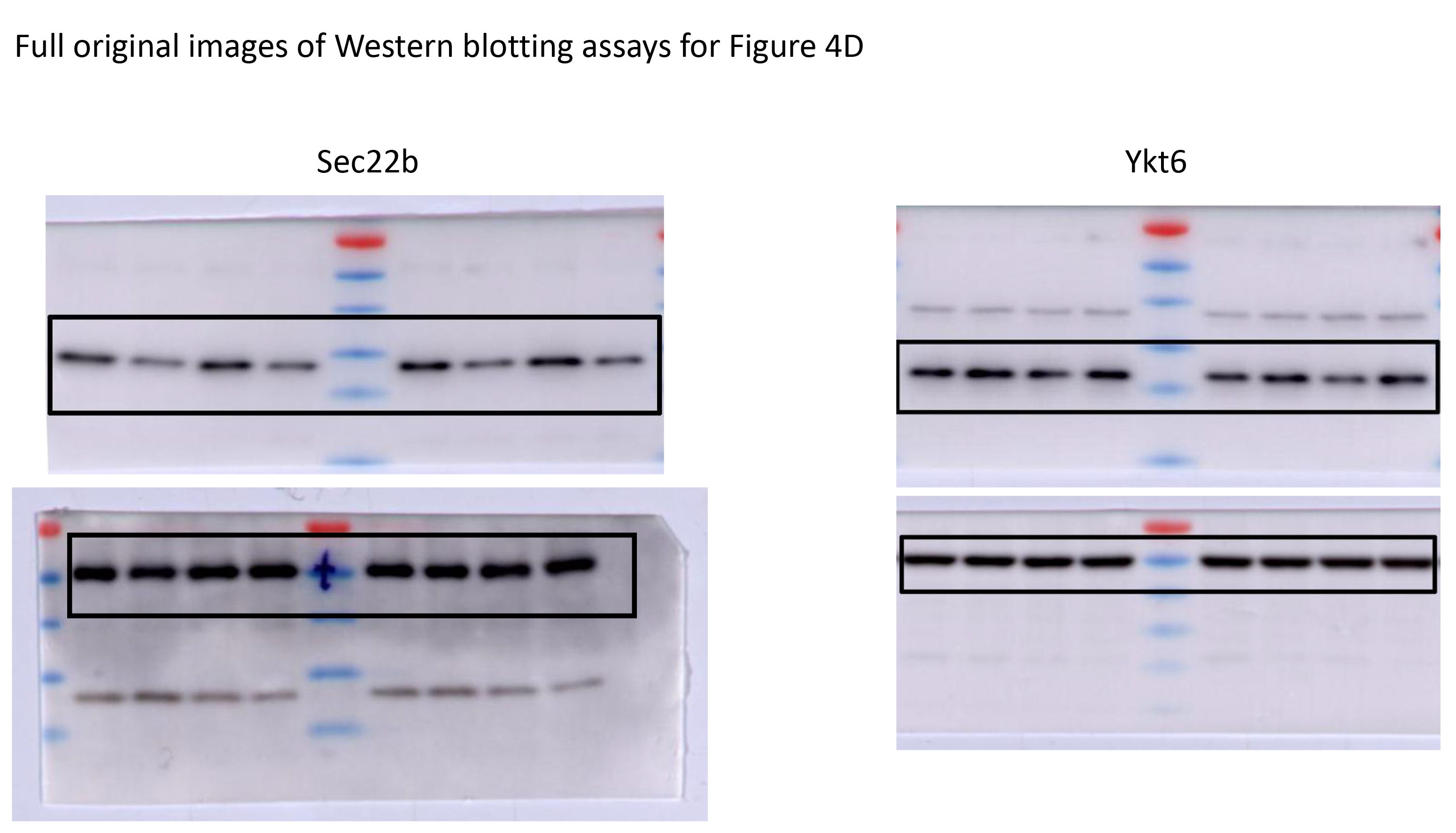

Supplement: Extended Data Figure 4-1 — Full original images of Western blotting assays for Figure 4. Download Figure 4-1, TIF file. [file ns-JN-RM-2030-21-s08.tif]

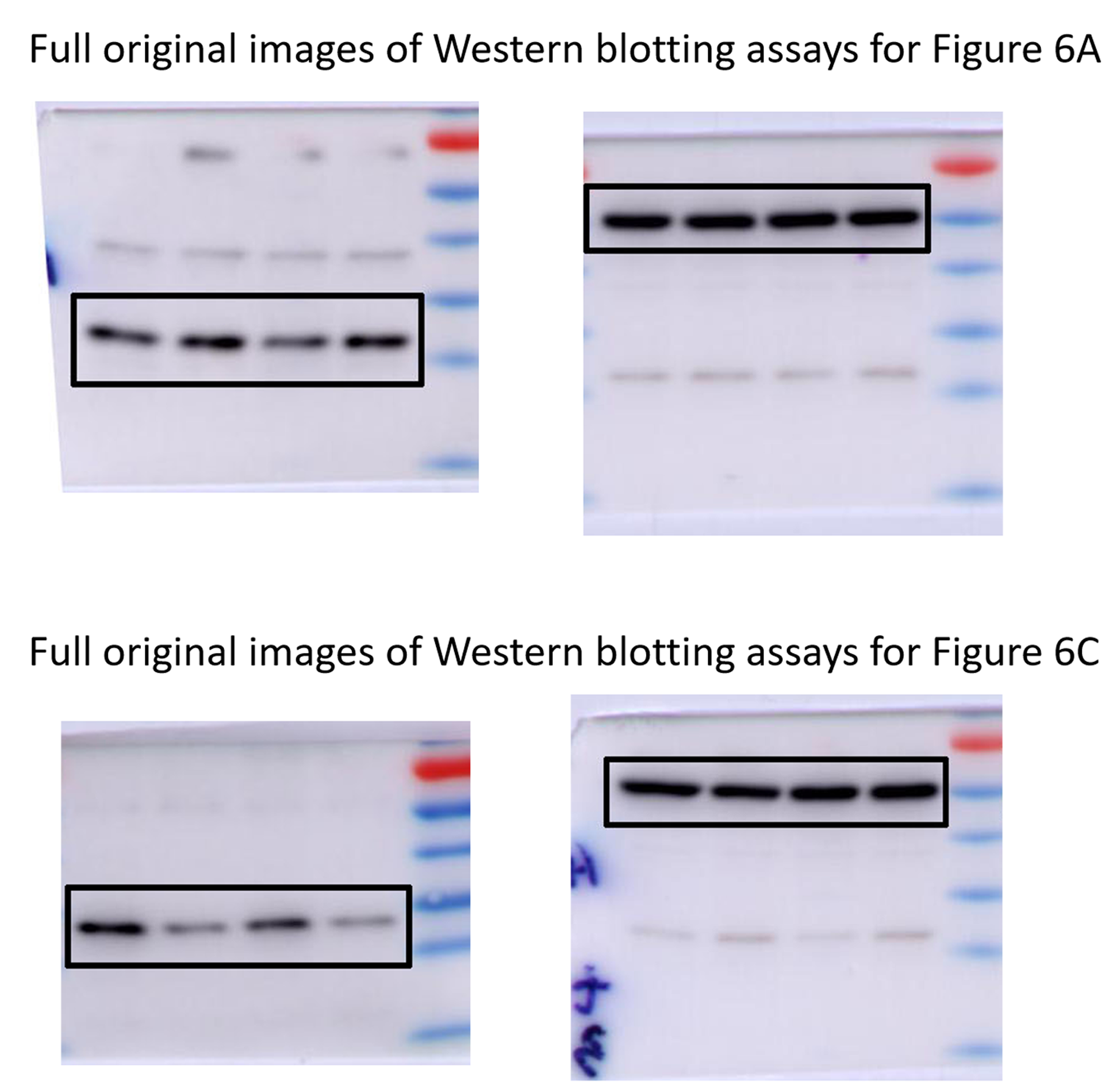

Supplement: Extended Data Figure 6-1 — Full original images of Western blotting assays for Figure 6. Download Figure 6-1, TIF file. [file ns-JN-RM-2030-21-s09.tif]
